# A bacterial genome in transition - an exceptional enrichment of IS elements but lack of evidence for recent transposition in the symbiont *Amoebophilus asiaticus*

**DOI:** 10.1186/1471-2148-11-270

**Published:** 2011-09-26

**Authors:** Stephan Schmitz-Esser, Thomas Penz, Anja Spang, Matthias Horn

**Affiliations:** 1 Department of Microbial Ecology, University of Vienna, Althanstrasse 14, 1090 Vienna, Austria; 2 Department of Genetics in Ecology, University of Vienna, Althanstrasse 14, 1090 Vienna, Austria; 3 Institute for Milk Hygiene, University of Veterinary Medicine Vienna Veterinärplatz 1, 1210 Vienna, Austria

**Keywords:** insertion sequence element, endosymbiont, *Bacteroidetes*, genome evolution

## Abstract

**Background:**

Insertion sequence (IS) elements are important mediators of genome plasticity and are widespread among bacterial and archaeal genomes. The 1.88 Mbp genome of the obligate intracellular amoeba symbiont *Amoebophilus asiaticus *contains an unusually large number of transposase genes (n = 354; 23% of all genes).

**Results:**

The transposase genes in the *A. asiaticus *genome can be assigned to 16 different IS elements termed ISCaa1 to ISCaa16, which are represented by 2 to 24 full-length copies, respectively. Despite this high IS element load, the *A. asiaticus *genome displays a GC skew pattern typical for most bacterial genomes, indicating that no major rearrangements have occurred recently. Additionally, the high sequence divergence of some IS elements, the high number of truncated IS element copies (n = 143), as well as the absence of direct repeats in most IS elements suggest that the IS elements of *A. asiaticus *are transpositionally inactive. Although we could show transcription of 13 IS elements, we did not find experimental evidence for transpositional activity, corroborating our results from sequence analyses. However, we detected contiguous transcripts between IS elements and their downstream genes at nine loci in the *A. asiaticus *genome, indicating that some IS elements influence the transcription of downstream genes, some of which might be important for host cell interaction.

**Conclusions:**

Taken together, the IS elements in the *A. asiaticus *genome are currently in the process of degradation and largely represent reflections of the evolutionary past of *A. asiaticus *in which its genome was shaped by their activity.

## Background

Mobile genetic elements such as phages, plasmids and transposable elements play a vital role in horizontal gene transfer and genome rearrangement in bacteria and archaea [1]. Among transposable elements, insertion sequence (IS) elements are particularly widespread within bacterial and archaeal genomes, and are considered the most abundant and ubiquitous genes in nature [2-6]. IS elements can have profound effects on chromosome structure and evolution. Due to their ability to disrupt genes and to induce rearrangements such as inversions, duplications and deletions they are key mediators of genome plasticity [2,3,7-9]. Although IS elements are perceived primarily as genomic parasites, their activity can also be beneficial. As composite transposons IS elements are able to mobilize adjacent genes, thereby mediating the spread of antibiotic resistance genes and genes involved in the catabolism of complex xenobiotics [10,11]. IS elements may also promote adaptation of their host genomes as demonstrated in experimental evolution experiments [12-15]. In addition, IS elements can influence or activate the expression of adjacent genes, e.g. by forming hybrid or fusion promoters or by containing outward-directed promoters [16-22].

IS elements are usually less than 2.5 kbp in length and have a relatively simple genetic organization. Most IS elements are flanked both by inverted and direct repeats and generally encode no function other than those involved in mobility, which is mediated by transposases [16]. IS elements have been classified into several families based on the degree of sequence conservation of their transposases and its catalytic site, similar genetic organization such as size, number of open reading frames (ORFs) and potential coding sequences (CDSs), inverted repeats, and genome target sites [2,16]. The majority of IS elements encode transposases containing the so-called DDE-motif consisting of the three amino acids aspartic acid, aspartic acid, and glutamic acid. These residues form the catalytic triad necessary for transposition. They are found in three regions (N2, N3, and C1) of the transposase amino acid sequence separated by spacers of various lengths [2,16].

Although IS elements are found in the majority of sequenced bacterial and archaeal genomes [2-4], their distribution is patchy, and their occurrence within single genomes is usually below 3% [2,6]. IS elements are very rare in the genomes of most ancient host-restricted symbionts or pathogens such as mutualistic insect and clam symbionts or chlamydiae [23,24]. On the other hand, elevated numbers of IS elements has been observed in the genomes of bacteria which adapted only recently to an intracellular or pathogenic lifestyle [8,25-27]. However, this view has been challenged by the recent detection of IS element-rich genomes in ancient symbionts such as *Wolbachia *spp. or *Orientia tsutsugamushi *[9,28-31]. Interestingly, the genomes containing the highest percentages of IS elements are from obligate intracellular bacteria: *Orientia tsutsugamushi *[28,29], the γ1 symbiont of the marine oligochaete *Olavius algarvensis *[32], the symbionts of grain weevils [26,33], and the amoeba symbiont *Amoebophilus asiaticus *5a2 [34].

*Amoebophilus asiaticus *is a Gram-negative, obligate intracellular symbiont, which has been discovered within an amoeba isolated from alkaline lake sediment [35]. Highly similar *A. asiaticus *strains have been recovered from various sources worldwide [35-38]. *A. asiaticus *shows highest 16S rRNA similarity to '*Candidatus *Cardinium hertigii', an obligate intracellular parasite of arthropods able to manipulate the reproduction of its hosts [39]. Both organisms belong to the phylum *Bacteroidetes *and form a monophyletic lineage in 16S rRNA-based phylogenetic trees [35], consisting only of symbionts and sequences retrieved from coral samples [40]. The *A. asiaticus *genome is only moderately reduced in size compared to many other obligate intracellular bacteria [41,42] but nevertheless, its biosynthetic capabilities are extremely limited [34]. The *A. asiaticus *genome encodes a hitherto unparalleled high number of proteins with eukaryotic domains such as ankyrin repeats, TPR/SEL1 repeats, leucine-rich repeats and domains from the eukaryotic ubiquitin system, and it contains an unusually large number of transposase genes (n = 354) corresponding to 23% of all genes [34].

Here, we report on the in-depth analysis of the IS elements in the *A. asiaticus *genome. We classified them and describe their main characteristics. We demonstrated that other symbionts closely related to *A. asiaticus *contain highly similar IS elements, and we could show that although they are transcribed, they exhibited no transpositional acitivity on a population level during a time period of almost 1,000 days. Taking into account evidence that no major rearrangements have occurred recently in the *A. asiaticus *genome, this suggests that the IS elements are evolutionary older components of the *A. asiaticus *genome, which likely played an important role during genome reduction and adaptation to an obligate intracellular life style.

## Results

### Diversity of IS elements in the *A. asiaticus *5a2 genome

IS elements make up 183 kbp (10%) of the *A. asiaticus *genome. In total, 354 transposase genes (corresponding to 23% of all CDSs) were identified in the detailed and manually curated analysis performed here (Tables 1, 2). Compared to other sequenced prokaryotic genomes, the percentage of IS elements as well as the number of IS elements per megabase genome is among the highest in *A. asiaticus *(Additional file 1, Figures S1, S2). We were able to assign the vast majority of these transposase genes (n = 329, 93%; including partial IS element copies) to 16 different IS elements (ISCaa1 to ISCaa16), which belong to eight different IS element families, with IS5 family IS elements being most abundant in the *A. asiaticus *genome (Table 2). Each of the 16 IS elements is present in 2 to 24 full-length copies in the *A. asiaticus *genome, the only exception being ISCaa1, which was identified earlier by the ISFinder website [43] and is only present as a single full-length copy (Table 2). This results in a total copy number of 122 full-length IS elements that are evenly spread across the *A. asiaticus *genome [34]. A high number of IS elements in *A. asiaticus *is truncated (n = 143), and in some cases (e.g. ISCaa5, ISCaa6 and ISCaa11) there are more truncated than full-length copies present (Table 2). Truncation sites were generally not conserved, i.e. truncations occurred in different regions, and truncated IS elements show varying lengths (Additional file 1, Figure S3). For most of the full-length IS element copies (n = 101, 83%) we could not identify direct repeats (Tables 1, 2). In the following sections we shortly describe few selected IS elements of *A. asiaticus *in more detail.

**Table 1:** IS element statistics for the genome of *A. asiaticus*

No. of protein coding genes	1557
No. of transposase encoding genes	354 (23% of all protein coding genes)

No. of transposase encoding genes assigned to IS elements	329 (93% of all transposase genes)

No. of full-length IS element copies*	122

No. of partial IS element copies*	143

No. of full-length IS element copies with functional transposase gene	106 (87% of all full-length IS element copies)

No. of full-length IS element copies without direct repeats	101 (83% of all full-length IS element copies)

* Note that IS elements can consist of more than one transposase gene

**Table 2:** IS elements in the *A. asiaticus *5a2 genome

IS element	IS family	Number of ORFs (predicted translational frameshift)	Length of IS element [bp]	Inverted repeats [bp]^a^	Direct repeats [bp]^b^	G+C content of IS element [%] (range)	Length of transposase [amino acids]	Number of full-length IS element copies (conservation on DNA level)	Number of partial IS element copies	Number of full-length IS element copies with intact transposase genes (conservation on protein level)
**ISCaa1^c^**	IS1	1	759	17/21	0	35.7	232	1	6	1

**ISCaa2**	IS5, ISL2 group	2 (-1)	916	19/20	0	36.3	275	3 (99-100%)	3	3 (99-100%)

**ISCaa3**	IS5 ISL2 group	2 (-1)	914	22/23	0	36.5	275	10 (99-100%)	5	9 (99-100%)

**ISCaa4**	IS1	2 (-1)	732	17/22	8/10	37.4 (36.2 - 38.0)	226	24 (85-100%)	8	21 (96-100%)

**ISCaa5**	IS982	1	932	18/21	0	38.2	274	10 (99-100%)	24	8 (99-100%)

**ISCaa6**	IS5, ISL2 group	1	991	15/19	0	36.6 (35.3 - 36.6)	275	18 (86-100%)	41	17 (88-100%)

**ISCaa7**	IS110	1	1483	0	0	31.9	343	3 (100%)	4	3 (100%)

**ISCaa8**	IS5, IS1031 group	2 (+1)	893	18/22	0	39.5	264	6 (99-100%)	6	6 (99-100%)

**ISCaa9**	IS5	3 (-1 ORFBC)	881	18/21	0	38.8	253	15 (100%)	3	15 (100%)

**ISCaa10**	IS200/IS605 IS200 group	1	527	0	0	38.5	147	7 (99-100%)	2	7 (99-100%)

**ISCaa11**	IS481	1	1031	10/11	6/3	38.9 (37.4 - 39.7)	314	10 (83-100%)	27	3 (87-100%)

**ISCaa12**	IS481	1	1210	29/34	6/2	37.6	364	3 (100%)	3	3 (100%)

**ISCaa13**	IS5, IS427 group	2 (+1)	860	17/21	0	40.7	253	2 (99%)	7	1

**ISCaa14**	IS110	1	1256	0	0	38.1	326	2 (97%)	0	1

**ISCaa15**	IS1182	1	1434	18/18	4/2	35.9	457	3 (100%)	3	3 (100%)

**ISCaa16**	IS6	1	837	15/18	0	37.4 (34.4 - 37.4)	235	5 (82-100%)	1	5 (85-100%)

^a ^number of base pairs conserved between left and right end repeats/length of the repeat

^b ^length of direct repeat/number of isoforms with direct repeat

^c ^ISCaa1 was identified by the ISFinder website http://www-is.biotoul.fr/})

### ISCaa4

**Figure 1:**
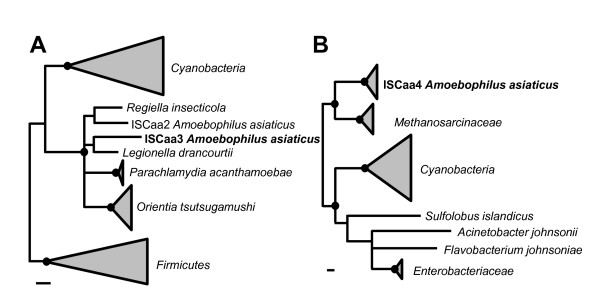
**Phylogenetic relationships of ISCaa3, IsCaa4 and related IS5 and IS1 family transposases**. Amino acid-based phylogenetic trees calculated with ARB using the TREE-PUZZLE algorithm are shown: **(A) **Phylogenetic relationships of ISCaa3 (IS5 family) and **(B) **ISCaa4 (IS1 family). Transposase sequences consisting of two ORFs were merged into a single ORF. Filled Black circles indicate nodes which are supported by TREE-PUZZLE support values and maximum parsimony bootstrap values (1000x resampling) greater than 90%. The bar represents 10% estimated evolutionary distance. Detailed versions of both phylogenetic trees are available as Additional file 1, Figure S5 and S7.

ISCaa4 is the most abundant IS element in *A. asiaticus*. It is present in 24 full-length copies, 21 of these copies should be able to produce an intact, functional transposase. ISCaa4 belongs to the IS1 family and shows a typical IS1 family DDE-motif [2,44]. Similar to other IS1 family members, the ISCaa4 transposase is encoded by two overlapping ORFs, which are probably translated into a 226 amino acid transposase by -1 ribosomal frameshifting (Table 2, Additional file 1, Figure S4). Translational frameshifting is often found in IS elements and represents an important mechanism regulating the expression of the transposases at a translational level [16,45]. Translation starts at the first ORF (*orfA*) and shifts to the -1 reading frame at the so-called slippery site and continues in a second overlapping ORF (*orfB*) resulting in a transframe ORFAB protein. The predicted frameshift site in ISCaa4 (AAAAAAG) is highly shift-prone in bacteria such as *A. asiaticus *that have only a single tRNA^Lys ^(anticodon: UUU) and lack the tRNA^Lys ^with the anticodon UUC [45,46]. In ISCaa4 five nucleotides downstream of the putative slippery site a stem-loop structure is predicted (ΔG -6.3 kcal/mol) (Additional file 1, Figure S4). Such stem-loop structures have been shown to be stimulatory for -1 ribosomal frameshifting [45,46]. Interestingly, ISCaa4 shows highest amino acid sequence identity (46 to 51%) to uncharacterized IS elements from methanogenic archaea of the family *Methanosarcinaceae*; the similarity to other transposases is lower than 40%. In phylogenetic trees, ISCaa4 forms a stable monophyletic group with these archaeal transposases, indicating interdomain horizontal gene transfer between methanogenic archaea and *A. asiaticus *(Figure 1, Additional file 1, Figure S5).

### ISCaa3

ISCaa3 is present in ten full-length copies in *A. asiaticus *and belongs to ISL2 group within the IS5 family (based on the presence of a typical DDE-motif) whose transposases typically consist of a single ORF [2,16]. The transposase of ISCaa3 however, is encoded by two overlapping ORFs most likely translated into a 275 amino acid protein by -1 ribosomal frameshifting. In contrast to other IS elements with canonical slippery sites like ISCaa4 and ISCaa9, no stimulatory stem-loop structure possibly enhancing ribosomal frameshifting is predicted downstream of the slippery site in ISCaa3 (Additional file 1, Figure S6). ISCaa3 shows highest amino acid sequence identity (57 to 66%) to ISCaa2 and IS elements found in the intracellular bacteria *Orientia tsutsugamushi*, *Legionella drancourtii*, *Regiella insecticola*, and *Parachlamydia acanthamoebae*. In phylogenetic analyses ISCaa2, ISCaa3 and related IS elements from intracellular bacteria consistently group together in all treeing methods applied, suggesting horizontal transfer of IS elements between these intracellular bacteria (Figure 1, Additional file 1, Figure S7). Interestingly, a number of cyanobacterial IS elements form a sister group with the ISCaa3-related IS elements.

### ISCaa9

ISCaa9 is an 881 bp IS element which is present in 15 almost identical copies (the differences occur only in the inverted repeats). ISCaa9 belongs to the IS5 family and shows highest amino acid sequence identity (45%) to ISMac15 from *Methanosarcina acetivorans *C2A, and 40% amino acid identity to ISWpi1, an IS element found in many *Wolbachia *strains [47,48]. The ISCaa9 transposase is encoded by three consecutive and overlapping ORFs which are translated into a 253 amino acid protein (Table 2, Additional file 1, Figure S8). We propose a stop codon read-through to occur at the stop codon (UGA) at nucleotide positions 263 to 265, which is supported by the presence of the stop codon in all 15 ISCaa9 copies in *A. asiaticus*, the absence of a stem-loop structure indicative of a terminator downstream of the stop codon, and the observation that UGAA is a weak stop codon quartet [49,50]. We predict that the stop codon is recoded into tryptophane (UGG), a common feature of UGAA stop codon quartets [50]. In addition, the majority of ISCaa9-related transposases encodes a tryptophane at the position of the stop codon read-through in ISCaa9 (Additional file 1, Figure S9). We predict a translational -1 frameshifting at a slippery site (AAAAAAG) between *orfB *and *orfC *(Additional file 1, Figure S7). Five nucleotides downstream of this putative slippery site, a stem-loop structure (ΔG -12.6 kcal/mol) is predicted in ISCaa9 (Additional file 1, Figure S8). The ISCaa9 transposase contains a DDE-motif, which is most similar to the IS1031 group within the IS5 family, the transposases of this group however, are usually encoded by a single ORF [2].

### ISCaa10

ISCaa10 is with a length of 527 bp a very short IS element that contains a single ORF encoding a 147 amino acid transposase. It belongs to the IS200/IS605 family and IS200 group of IS elements comprising the shortest known transposases [2]. Members of the IS200 group are unusual IS elements because their transposases do not contain the DDE-motif found in most transposases. Instead they belong to the Y1 transposases with a catalytic tyrosine residue and a conserved HuH motif (consisting of a histidine, a hydrophobic amino acid, and another histidine) [51,52]. Interestingly, this motif is present only in two of seven ISCaa10 copies; in the others, the second histidine is replaced by tyrosine, which might render these copies nonfunctional. Other unusual features of IS200 IS elements, that are also found in ISCaa10, are the absence of both direct and terminal inverted repeats and the presence of secondary structures leading to low transcriptional and transpositional activity [51-53]. For example, IS200 from *Salmonella typhimurium *LT2 forms two stem-loop structures: The first is a transcriptional repressor terminating impinging transcripts, the second acts at the translational level and occludes the ribosome binding site [53]. Similarly, a stem-loop structure is predicted ten nucleotides upstream of the start codon of the ISCaa10 transposase and close to the 3' end of ISCaa10 (ΔG -13 kcal/mol and -20.3 kcal/mol, respectively). ISCaa10 shows highest amino acid sequence identity (76%) to (uncharacterized) IS200 family transposases from *Xenorhabdus nematophila *(GenBank accession no: YP_003712757).

### Unclassified IS elements

Twenty-five transposase genes could not be assigned to either of the 16 *A. asiaticus *IS elements under the criteria applied here. Among these unclassified full-length transposases two transposases belong to the IS110 family (Aasi_1379 and Aasi_1284); and to the Tn3 family (Aasi_0096, Aasi_0545); one belongs to the IS3 family (probably consisting of the two consecutive ORFs Aasi_1748 and Aasi_0907); and two belong to the YhgA-like family of putative transposases (Aasi_0894, Aasi_1306; PFAM-family PF04754).

### Conservation of IS elements among different *A. asiaticus *strains

In order to analyze whether the IS elements found in the genome of *A. asiaticus *5a2 are also present in closely related *A. asiaticus *strains, we performed PCR using primers targeting the 13 most abundant IS elements (Additional file 2, Table S1) with genomic DNA from *A. asiaticus *strain EIDS3 [35] as well as from two novel *A. asiaticus *isolates, *A. asiaticus *US1 and *A. asiaticus *WR. These strains show 98.9%, 99.2%, and 98.5% 16S rRNA sequence similarity to *A. asiaticus *5a2, respectively, corresponding to strain and species level diversity, respectively. Six out of the 13 IS elements analyzed here were detected in all four *A. asiaticus *isolates. Cloning and sequencing of PCR products obtained from *A. asiaticus *EIDS3 revealed nucleic and amino acid sequence identities to consensus sequences of the *A. asiaticus *5a2 IS elements of 87% to 98% (Table 3). The lack of PCR products for some IS elements indicates either the absence of these IS elements in the investigated *A. asiaticus *strains or a low degree of conservation and hence the absence of or mismatches with the primer target sites.

**Table 3:** Occurrence of IS elements in four different *A. asiaticus *strains based on PCR.

IS element in *A. asiaticus *5a2	*A. asiaticus *EIDS3 (amino acid identity to *A. asiaticus *5a2 element)	*A. asiaticus *WR	*A. asiaticus *US1
ISCaa2	+ >(95%)	-	-

ISCaa3	+ >(97%)	+	+

ISCaa4	-	+	-

ISCaa5	+ >(94%)	-	+

ISCaa6	+ >(92%)	-	+

ISCaa7	-	-	-

ISCaa8	+ >(91%)	+	+

ISCaa9	+ >(94%)	+	+

ISCaa10	+ >(98%)	+	+

ISCaa11	+ >(90%)	+	+

ISCaa12	+ >(98%)	+	+

ISCaa15	+ >(87%)	+	-

ISCaa16	-	-	-

### Transcription but lack of transpositional activity of the *A. asiaticus *IS elements

**Figure 2:**
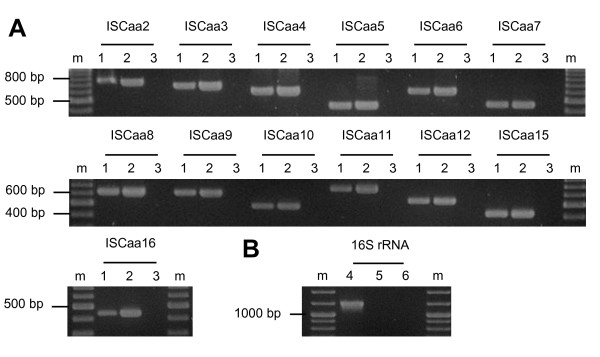
**Transcription of IS elements during intracellular growth of *A. asiaticus *5a2 in its *Acanthamoeba *host**. Transcription of 13 selected *A. asiaticus *IS elements was analyzed with reverse transcriptase PCR. Whole RNA from the *Acanthamoeba *host harboring *A. asiaticus *was transcribed into cDNA and subsequently used for PCR. **(A) **Reverse transcriptase PCR reactions. Lanes 1: cDNA; lanes 2: positive control, genomic DNA purified from amoebae containing *A. asiaticus*; lanes 3: negative control, no nucleic acids added. **(B) **PCR using 16S rRNA gene-specific primers was used to control for the absence of DNA in the RNA preparation. Lane 4: positive control, genomic DNA; lane 5: RNA; lane 6: negative control, no nucleic acids added. m: molecular size marker. Reverse transcriptase-PCR reactions were performed in three biological independent replicates.

**Figure 3:**
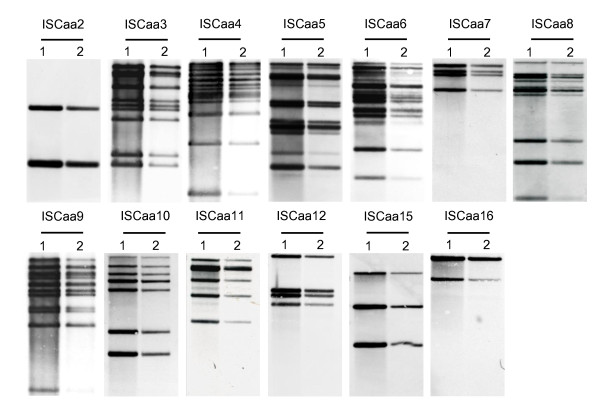
**Analysis of transpositional activity of the most abundant IS elements of *A. asiaticus *5a2**. Transposition of IS elements was analyzed with Southern hybridizations using IS element-specific probes and DNA purified from the same *A. asiaticus *5a2 culture in November 2006 and July 2009, respectively. DNA was digested with Eco32I (except for ISCaa2, where HindIII was used). Each visible band corresponds to at least one IS element copy on the respective DNA fragment, as the restriction endonucleases do not cut within the IS elements. IS elements are indicated above each hybridization; lanes 1: DNA isolated November 2006; lanes 2: DNA isolated July 2009. The absence of changes in the banding patterns between both time points indicates that no (major) chromosomal rearrangements due to IS element transposition has occurred.

The large copy number and the high degree of conservation of some IS elements identified in the *A. asiaticus *5a2 genome might indicate that they are transpositionally active. To investigate this, we first asked whether the IS elements are transcribed during intracellular replication of *A. asiaticus *in its amoeba host. Using reverse transcriptase (RT)-PCR, we analyzed the transcription of those 13 IS elements that are present in at least three copies in the genome (ISCaa2 to ISCaa12, ISCaa15 and ISCaa16). The detection of transcripts of all 13 IS elements demonstrates that at least one copy each is actively transcribed (Figure 2). Next, we used Southern hybridizations to check for chromosomal rearrangements resulting from transposition events [12,14,54]. We analyzed the same 13 IS elements for which we could show transcription and compared DNA from the same *A. asiaticus *culture isolated in November 2006 and in July 2009, respectively, a period of 984 days. We could not detect differences in the banding pattern indicative for chromosomal rearrangements in Southern hybridizations for any of the IS elements tested (Figure 3).

### Contiguous transcription of IS elements and their downstream genes

**Figure 4:**
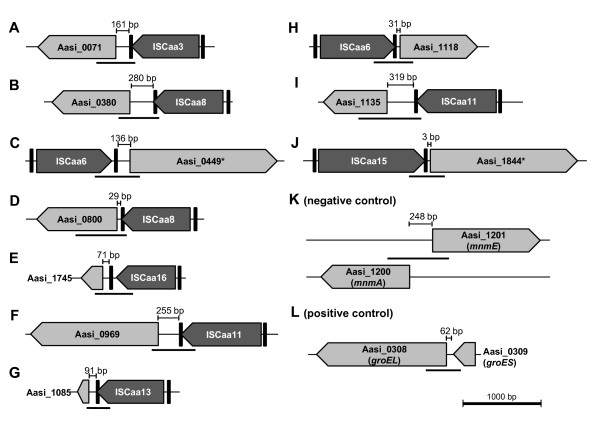
**Genomic organization of selected *A. asiaticus *IS elements and their downstream genes**. IS elements are shown as dark grey pentagons; downstream genes as light grey pentagons; inverted repeats are represented by vertical black bars. The distance between IS elements and downstream genes is indicated. Reverse transcriptase PCR was used to test for contiguous transcription (Figure 5); the size of the expected PCR products are indicated as horizontal black lines below each locus. Panels (**A**) to (**J**) show the organization of IS elements and their downstream genes. Panels (**K**) and (**L**) show the genomic organization of genes used for control reactions in the reverse transcriptase PCR experiments. Aasi_1200 and Aasi_1201 are located in opposite direction on different strands and served as negative control; Aasi_0308 and Aasi_0309 representing the *groEL*/*groES *operon served as positive control. An asterisk (*) indicates genes which are not drawn to scale (due to their length). Further details on the genomic organization of the analyzed loci and the downstream genes (including locus_tags of the IS elements) are available in Additional file 2, Table S3.

**Figure 5:**
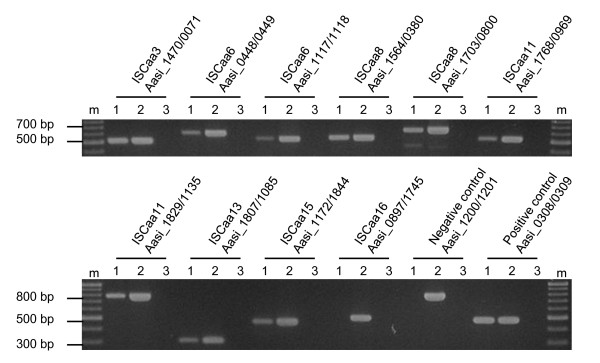
**Contiguous transcription of *A. asiaticus *IS elements with their downstream genes**. Transcription was analyzed with reverse transcriptase PCR. Whole RNA from the *Acanthamoeba *host and *A. asiaticus *5a2 was transcribed into cDNA and subsequently used for PCR. Genomic organization of the tested loci and the expected sizes of the PCR products are shown in Figure 4. Locus_tags are indicated above the gel images; lanes 1: cDNA; lanes 2: positive control, genomic DNA; lanes 3: negative control, no nucleic acids added. Contiguous transcription was demonstrated for all tested loci except for the IS element Aasi_0897 (ISCaa16) and its downstream gene Aasi_1745. The genes Aasi_1200/1201 were used as negative control (as they are located on different DNA strands and have opposing orientation). The *groEL/groES *operon (Aasi_0308/0309) was used as a positive control. All experiments were performed in three biological independent replicates.

Some of the *A. asiaticus *IS elements are in close proximity to their downstream genes (with distances less than 50 bp). As previous reports have shown that IS elements can influence the transcription of neighboring genes [17-21], we investigated whether contiguous transcripts between *A. asiaticus *IS elements and downstream genes occur. We analyzed ten selected loci where IS elements and their downstream genes are encoded on the same strand and have the same orientation (Figure 4). Using RT-PCR we could show contiguous transcripts of the investigated IS elements with their downstream genes at 9 out of 10 analyzed loci (Figure 5). We performed two control experiments in order to exclude that the observed transcripts from RT-PCR derive from unspecific background noise transcriptional read-through. One control targeted an unlikely contiguous transcript between two genes located on different strands and oriented in opposite directions (Aasi_1200/1201, Figure 4). We could not detect transcripts in this control reaction (Figure 5), indicating that the observed transcripts from the nine loci of IS elements and their downstream genes are above unspecific read-through transcription. This is further supported by a second, semi-quantitative control experiment in which we compared RT-PCR products (using the same conditions) from contiguous transcripts between IS elements and their downstream genes with the products from RT-PCR reactions targeting only the downstream genes (Additional file 1, Figure S10). In all cases the obtained bands were of similar intensity, providing further evidence that the observed contiguous transcripts are above unspecific transcriptional read-through.

## Discussion

Mobile genetic elements such as IS elements move within and between genomes. Owing to its intracellular lifestyle in free-living amoebae *A. asiaticus *is, however, largely shielded from other bacteria. Although horizontal gene transfer seems unlikely to occur under these circumstances, previous studies proposed that amoebae may serve as hot spots for horizontal gene transfer among intracellular bacteria [34,55], and according to the 'intracellular arena' hypothesis genetic material may move in and out of communities of obligate intracellular bacteria that co-infect the same intracellular host environment [23]. We identified four IS elements in *A. asiaticus *that were likely involved in horizontal gene transfer although the direction of the transfer cannot be inferred (ISCaa2, ISCaa3, ISCaa4, ISCaa12; Figure 1, Additional file 1, Figure S5, S7). Three of these IS elements group with IS elements from several other intracellular bacteria related to rickettsiae, legionellae and chlamydiae, and consistent with previous findings it is conceivable that amoebae or other protozoa served as a common habitat for these microbes. One IS element of *A. asiaticus *is most closely related to IS elements found in free-living methanogenic archaea (*Methanosarcinaceae*). Anoxic aquatic sediments, where free-living amoebae and methanogenic archaea can be found, might represent a possible shared habitat facilitating horizontal gene transfer [56-58]. Horizontal gene transfer of IS elements between distantly related organisms is rather rare [59]. Hence the discovery of related IS elements in three different bacterial phyla (*Bacteroidetes, Proteobacteria*, *Chlamydiae*) and the *Archaea *might be surprising. However, a recent study based on the analysis of 800 bacterial and archaeal genomes showed that although the majority of horizontal gene transfer events occur between closely related organisms there is a considerable number of large-distance horizontal gene transfer events [60]. Our observations expand our view on the extent of horizontal gene transfer of IS elements among distantly related microbes, and they provide a glimpse into past interactions of *A. asiaticus *with other microbes during its evolutionary history.

Several lines of evidence point to an ancient origin of many IS elements in *A. asiaticus*. First, ISCaa4, ISCaa6, ISCaa11, and ISCaa16, which together make up 46% of all full-length *A. asiaticus *IS elements, show a remarkably low degree of sequence conservation among their different copies (Table 2). This is in contrast to high sequence similarities expected if IS elements have entered a genome and spread only recently [4,61]. Second, the high number (n = 143) of truncated IS element copies suggests that these IS elements have been present in the *A. asiaticus *genome for extended time periods during which they disintegrated slowly. Third, the GC-content of the *A. asiaticus *IS elements (37.3% on average, range: 31.9 to 40.7%) is similar to the overall GC content of the *A. asiaticus *genome (35.0%). This suggests that considerable time has elapsed to allow the base composition of the IS elements to adapt towards the general base composition of the *A. asiaticus *genome [62]. Finally, at least six IS elements are conserved among four different *A. asiaticus *strains, some of which show a relatively high divergence (Table 3). Taking into account that our PCR based screening likely underestimates the actual number of shared IS elements (due to mismatches at the primer binding sites in more diverged homologs), this indicates that many - if not most - *A. asiaticus *IS elements were already present in the last common ancestor of the *A. asiaticus *strains investigated here. Taken together, there is compelling evidence that the IS elements have been residing in the *A. asiaticus *genome for considerable evolutionary time periods.

We noted previously that the *A. asiaticus *genome shows a GC skew pattern typical for most bacterial genomes with two major shifts at the origin and terminus of replication and only few local deviations, which are indicative of recent genome rearrangements [34]. This is remarkable because with the exception of *Lactobacillus helveticus *DPC 4571 and *Shigella sonnei *Ss046 (whose genomes contain significantly lower percentages of IS elements than *A. asiaticus*; Additional file 1, Figures S1, S2)[27,63], all other bacteria with high numbers of IS elements do not show such a regular genomic GC skew pattern (Additional file 1, Figure S11). Thus, despite of the high number of IS elements, the *A. asiaticus *genome has not been reshuffled extensively recently, which indicates that most IS elements are transpositionally inactive and also that recombination events between highly similar IS element copies have not occurred.

A mechanism by which apparently inactive, non-functional IS elements can be maintained in bacterial genomes is gene conversion, which was described recently for the genome of *Wolbachia *wBm, a mutualistic symbiont of the nematode *Brugia malayi *whose genome contains a number of highly similar IS element copies rendered non-functional by multiple stop codons and frame shifts [64,65]. In contrast to *Wolbachia *wBm, *A. asiaticus *still encodes intact copies of each IS element, and many IS elements show relatively high sequence divergence (Table 2). In addition, the transposase genes of different non-functional IS element copies show variable pseudogenization states. This largely rules out gene conversion as the main mechanism for maintenance of IS elements in *A. asiaticus*.

Rather unexpectedly, we detected transcription of 13 *A. asiaticus *5a2 IS elements during intracellular growth in amoebae (Figure 2). Generally, IS elements are among the lowest expressed genes due to their potentially detrimental effects on the host genome [16,61,66-70]. For several *A. asiaticus *IS elements (ISCaa2, ISCaa9, ISCaa10, ISCaa14, ISCaa15; data not shown) stable hairpin structures within the first 50 bp of the IS elements are predicted, which might interfere with expression both at the transcriptional and the translational level, thus controlling the activity of these IS elements. In addition, evidence for programmed translational frameshifting, another regulatory mechanism, can be found in six *A. asiaticus *IS elements (Table 2). Translational frameshifting acts at the level of translation elongation between two consecutive (and partially overlapping) open reading frames where the ribosome slides one basepair up- or downstream at a so-called slippery site [16,45,66]. For several IS elements of *A. asiaticus*, the occurrence of frameshifting is supported by the presence of a canonical slippery site, of stimulatory secondary structures downstream of the slippery site and, most importantly, the merged amino acid sequences of the IS elements transposase ORFs show more significant Blast hits than the single ORFs alone (data not shown). In summary, transcription of several IS elements occurs in *A. asiaticus*, but there is evidence that many IS elements are tightly regulated both at the transcriptional and the translational level.

Southern hybridizations demonstrated the absence of major transposition events and genome rearrangements for *A. asiaticus *during a time period of 984 days (Figure 3). With an estimated generation time of *Acanthamoeba *sp. 5a2 infected with *A. asiaticus *of 19 h (data not shown), this time period corresponds to approximately 1200 generations of the *Acanthamoeba *host. Although the generation time of *A. asiaticus *is unknown, it must be shorter than that of its amoeba host (due to the high number of symbionts per amoeba cell [35,37]). The analyzed time period thus corresponds to considerably more than 1200 *A. asiaticus *generations. For *E. coli *and *Lactococcus lactis*, the first IS element-mediated genomic changes (insertions, deletions, duplications) occurred already after 400 to 500 generations [12-15]. This indicates that the time period monitored in our study should be sufficient to detect IS element-mediated genomic rearrangements. However, in contrast to our experiment, in these studies bacterial cultures were exposed to environmental stress conditions with respect to nutrient availability, temperature, or oxygen, facilitating adaptive changes. Although no genome rearrangements were observed for *A. asiaticus*, we cannot exclude the possibility that transposition events occurred in individual *A. asiaticus *cells which subsequently became not fixed at the population level and would thus be undetectable by our experimental approach. However, Southern blot is a highly sensitive method [71], and we have estimated that we should be able to monitor changes in Southern blot patterns in subpopulations consisting of only a few to a few hundred of amoeba host cells (Additional file 2, Table S2). Taking into account typical densities of *Acanthamoeba *sp. 5a2 infected with *A. asiaticus *during in vitro cultivation of 10^5 ^up to 10^7 ^cells/ml, the sensitivity of our assay should thus be sufficient to detect variations even in very small subpopulations. The IS elements in *A. asiaticus *are therefore most likely transpositional inactive. Their abundance is explained by transpositional activity in the evolutionary past of *A. asiaticus*, and while still being transcriptionally active, most IS elements are transpositionally inactive in extant *A. asiaticus*. In addition to a tight transcriptional and (post-)translational control there are several other conceivable explanations for this observation. For example, *A. asiaticus *might lack host factors required for transposition activity of IS elements although most of those are specific for certain IS elements; they act at different steps and cellular processes and their exact role in transposition is still largely unclear [16,66,72]. Alternatively, the small, reduced genome of *A. asiaticus*, which is highly adapted to the intracellular life style and optimized for host cell interactions, might not allow for major rearrangements as most transposition events would be deleterious rendering the cell nonviable.

One reason why some IS elements were retained in the *A. asiaticus *genome despite of the apparent lack of transpositional activity might be their influence on the transcription of downstream genes. Indeed, we could show contiguous transcripts of IS elements with their downstream genes at 9 out of 10 tested loci (Figure 4, Figure 5). In some cases, the distance between the IS element and the start codon of the downstream gene is too short to include known *Bacteroidetes *Shine-Dalgarno sequences, which are located at -33 and -7 bp relative to the transcription initiation site [73,74]. Expression of the respective downstream genes might thus depend on promoter sequences located within the upstream IS element (e.g. in the inverted repeats), a feature often found in IS elements [16,66], or on the endogenous promoter of the IS element. In other cases the distance between the analyzed IS elements and their downstream genes was larger (up to 300 bp). Similar polycistronic mRNAs starting from IS elements including downstream genes have been described recently for two IS elements in *Francisella tularensis *[17] and in *Mycobacterium tuberculosis *IS6110 [21]. It is striking that many of the genes whose transcription is affected by the presence of IS elements in *A. asiaticus *likely play an important role (Additional file 2, Table S3). For example, Aasi_1844 is an uncharacterized membrane protein conserved among most *Bacteroidetes *and *Chlorobi*; Aasi_1118 contains six TPR/SEL1 repeats, eukaryotic domains that can be involved in host cell interaction [75], and Aasi_0380 is a ferritin homolog involved in iron storage. Furthermore, a genomic organization of IS elements and downstream genes similar to the loci analyzed in this study was found in 44 other regions on the *A. asiaticus *genome (data not shown), suggesting that contiguous transcripts between IS elements and downstream genes are even more widespread and represent a more general feature of *A. asiaticus*.

Genome reduction is an important process during the adaptation of bacteria to an obligate intracellular life style, and IS elements are considered to be important in this process [2,8]. The genome of *A. asiaticus *is only moderately reduced compared to other obligate intracellular bacteria [41,42]. Its genome size is with 1.9 Mbp notably larger than that of other related symbionts in the *Bacteroidetes *(0.2 to 1.1 Mbp), but smaller than those of free-living relatives (2.2 to 9.1 Mbp, Additional file 1, Figure S12). The genome of *A. asiaticus *thus represents a transitional stage in genome reduction. We argue that the IS elements in the *A. asiaticus *genome are evolutionary remnants. They have been present in the *A. asiaticus *genome for extended time periods and reflect the organism's evolutionary history. The IS elements proliferated and were important during the adaptation of *A. asiaticus *to the intracellular life style, but they became increasingly redundant. The *A. asiaticus *genome thus represents a snapshot of a bacterial genome which was shaped by the activity of IS elements but whose IS elements are largely inactive and in the process of further degradation at the present stage.

## Conclusion

Analysis and characterization of the *A. asiaticus *IS elements provides evidence for an extremely IS element-rich genome, which seems to be evolutionary surprisingly stable - a feature not found in other IS element-rich genomes. The presence of contiguous transcripts between IS elements and their downstream genes indicates that these IS elements influence the transcription of their downstream genes, most of which likely play an important role for *A. asiaticus*. Proliferation of IS elements in the evolutionary past of *A. asiaticus *might thus have been an important process during the adaptation of *A. asiaticus *to an intracellular life style in which its genome was shaped by their activity.

## Methods

### Sequence analyses

The genome sequence of *A. asiaticus *5a2 has recently been determined and analyzed [34] and is available at GenBank under accession no. CP001102. For identification of IS elements we first compiled a list of candidate transposase genes by keyword, PFAM and InterPro domain search available in the genome annotation software Pedant [76]. We then manually inspected this list in order to verify the evidence for each gene to encode a putative transposase. In addition, further transposase genes were identified by manually analyzing each predicted gene in the *A. asiaticus *genome (e.g by using Blast against the NCBI nr dataset (provided by the annotation software Pedant), Blast against the ISfinder database http://www-is.biotoul.fr/) In order to classify the transposases into groups of homologs we performed Blast (BlastP, BlastN) searches against the *A. asiaticus *genome. In order to identify full-length IS elements, the gene sequences of the transposases and surrounding genomic regions were aligned and the full-length IS elements were then manually identified based on these alignments. Partial IS element copies were identified by BlastN and BlastP searches and alignment of full-length IS element copies against the *A. asiaticus *genome. Inverted repeats were identified with the EMBOSS software palindrome and einverted [77]. Nucleic acid sequences of IS elements and amino acid sequences of transposase genes were aligned with MAFFT [78]; alignments were visualized using BOXSHADE http://www.ch.embnet.org/software/BOX_form.html. For detection of direct repeats the nucleic acid alignments of the IS elements and their genomic neighborhood were searched manually. We grouped and classified IS elements using Blast against the ISfinder website http://www-is.biotoul.fr/[43] and the following criteria: (i) a minimum amino acid sequence identity of 30% of the transposase to described transposases, (ii) the presence of flanking inverted repeats (exception: IS elements belonging to family IS110 and IS200/605, which do not have flanking inverted repeats), and (iii) the presence of at least two copies in the genome. IS element copies that shared more than 80% nucleic and amino acid sequence identity over at least 98% of their length were considered isoforms. The nomenclature suggested by the ISFinder website was used for naming of IS elements http://www-is.biotoul.fr/[43]. mRNA secondary structures were predicted using the Mfold web server [79]. For calculations of phylogenetic relationships of the transposases from selected IS elements, the amino acid sequences of overlapping ORFs were merged resulting in a single peptide sequence (in the case of IS elements with predicted ribosomal frameshifting), aligned with MAFFT [78] and imported into ARB [80]. Phylogenetic trees were constructed with the Phylip maximum parsimony, distance matrix (Fitch), ProML (using the JTT amino acid replacement model) methods and the TREE-PUZZLE algorithm (using the VT model of amino acid substitution) [81,82] implemented in ARB. Maximum parsimony bootstrap analysis was performed with 1000 resamplings. A filter considering only those alignment positions that were conserved in at least 10% of all sequences (resulting in a total number of 274 and 228 alignment columns for ISCaa3 and ISCaa4, respectively) was used for all treeing calculations. For each IS element analyzed, the overall tree topology between the different treeing methods applied was consistent, thus only trees calculated using the TREE-PUZZLE algorithm are shown.

### Cultivation and isolation of amoebae

Amoebae harboring *A. asiaticus *5a2 (ATCC no. PRA-228) and amoebae harboring *A. asiaticus *EIDS3 (ATCC no. PRA-221) were maintained as adherent culture in 25 cm^2 ^tissue culture flasks containing 10 ml peptone-yeast-glucose medium (PYG: 20 g/l proteose peptone, 2 g/l yeast extract, 90 mM glucose, 4 mM MgSO_4_*7H_2_O, 3.4 mM C_6_H_5_Na_3_O_7_*2H_2_O, 2.5 mM KH_2_PO_4_, 1.3 mM Na_2_HPO_4_*2H_2_O, 51 μM Fe(NH_4_)_2_(SO_4_)_2_*6H_2_O). Cultures were incubated at 27°C and passaged at confluency by 1:10 dilution of the culture every five to ten days. Amoebae harboring *A. asiaticus *WR and amoebae harboring *A. asiaticus *US1 were isolated from soil and lake sediment (Alkaline lake "Unterer Stinker", Burgenland, Austria) samples, respectively, using non-nutrient agar plates seeded with live or heat-inactivated *Escherichia coli *as described previously [83]. Both isolates were cultivated as described above using modified PYNFH (10 g/l bacteriological peptone, 10 g/l yeast extract, 1 g/l yeast nucleic acid, 15 mg/l folic acid, 1 mg/l hemin, 2.6 mM KH_2_PO_4_, 2,8 mM Na_2_HPO_4_*2H_2_O).

### DNA isolation

Amoebae harboring *A. asiaticus *5a2, EIDS3, WR and US1 were harvested by centrifugation (5000 × g, 10 min). The cell pellet was resuspended in 250 μl 1× TE buffer (10 mM Tris, 1 mM EDTA, pH 8) and subsequently used for high molecular weight DNA isolation using a modified protocol from Zhou et al. [84]. Briefly, 675 μl DNA extraction buffer (100 mM Tris/HCl, 100 mM EDTA, 100 mM sodium-phosphate, 1.5 M NaCl, 1% (w/v) cetyltrimethylammonium bromide (CTAB), 200 μg/ml proteinase K, pH 8.0) were added to the cell pellet and incubated for 30 min at 37°C. After addition of 75 μl 20% (w/v) SDS, the samples were incubated at 65°C for 1 h. To recover the aqueous phase, the lysate was mixed with an equal volume of chloroform/isoamylalcohol (24:1, v/v) and centrifuged (11200 × g, 10 min). Nucleic acids were precipitated with 0.6 volume isopropanol at room temperature for 1 h. The resulting pellet from centrifugation (16000 × g, 20 min) was washed with 70% ethanol, centrifuged again (16000 × g, 5 min), resuspended in ddH_2_O and stored at -20°C until use.

### Transcription analysis

Amoebae harboring *A. asiaticus *5a2 were harvested by centrifugation (7000 × g, 3 min, 27°C). The resulting cell pellet was resuspended in 750 μl TRIzol (Invitrogen Life Technologies), transferred to a Lysing Matrix A tube (MP Biomedicals) and homogenized using a BIO101/Savant FastPrep FP120 instrument (speed: 4.5 m/sec, 30 sec). RNA was extracted by phase separation, precipitation, washing and redissolving according to the recommendations of the manufacturer (TRIzol, Invitrogen Life Technologies). Remaining DNA was removed using the TURBO DNA-*free *Kit (Ambion). After DNase treatment RNA was resuspended in ddH_2_O_DEPC _and stored at -80°C until use. The absence of DNA contamination in the DNase-treated RNA was verified by performing a control PCR with 42 cycles using primers targeting the 16S rRNA gene of *A. asiaticus *5a2 (Additional file 2,Table S1). DNA-free total RNA (containing host and symbiont RNA) was used to synthesize cDNA using the RevertAid™ First Strand cDNA Synthesis Kit (Fermentas) according to the recommendations of the manufacturer. cDNA was subsequently used as template in standard PCR reactions (35 cycles and annealing temperatures according to the optimal conditions for the primers listed in Additional file 2, Table S1). Negative controls (no cDNA added) and positive controls (genomic DNA) were included in all PCR reactions. Amplification products were sequenced to ensure that amplification was specific. All experiments were performed in biologically independent triplicates.

### PCR screening for IS elements in different *A. asiaticus *strains

A standard PCR cycling program with 35 cycles at low stringency (annealing temperature 45°C) with primers specific for different *A. asiaticus *5a2 IS elements was used for the detection of IS elements in the *A. asiaticus *strains EIDS3, WR and US1 (see Additional file 2, Table S1 for primer sequences). Negative (no DNA added) and positive controls (genomic DNA from *A. asiaticus *5a2) were included in all PCR reactions. The amplified fragments from *A. asiaticus *EIDS3 were cloned using the TOPO TA cloning kit and cloning vector pCRII (Invitrogen Life Technologies). Nucleotide sequences of the cloned DNA fragments were determined on an ABI 3130 XL genetic analyzer using the BigDye Terminator kit v3.1 (Applied Biosystems).

### Southern hybridizations

Southern hybridization was performed using a modified protocol based on Sambrook et al. [71]. Two μg DNA (containing host amoeba and *A. asiaticus *DNA) were digested with Eco32I for all investigated IS elements, except for ISCaa2, for which DNA was digested with HindIII and subsequently separated on a 0.7% TAE agarose gel (4°C, 17 h, 30 V). The gel was depurinated for 10 min in 0.25 M HCl, denaturated for 30 min in 1.5 M NaCl/0.5 M NaOH and neutralized for 30 min in 1.5 M NaCl/1 M Tris-HCl (pH 7.5). Between each of these steps the gel was briefly rinsed in ddH_2_O. DNA was transferred onto Hybond N^+ ^nylon membranes (GE Healthcare) with a vacuum transfer system and 20× SSC (3M NaCl, 0.3M sodium citrate, pH 7.0) as transfer buffer for 30 min. After immobilizing the DNA by UV cross-linking (120000 μJ cm^-2^), the membrane was briefly rinsed in ddH_2_O. Pre-hybridization was carried out for 2 h at 42°C in hybridization buffer (containing 50% formamide, 5× SSC, 2% blocking reagent (Roche), 0.1% N-lauroyl sarcosyl sodium salt, 0.02% SDS (v/v)) in a rotation hybridization chamber as the following steps. The blot was hybridized with digoxygenin (DIG)-labeled probes (synthesized using the PCR DIG Probe Synthesis Kit, Roche; each probe was specific for a single IS element; see Additional file 2, Table S1) and hybridization buffer over night at 42°C. The membrane was washed twice for 15 min each with 2× SSC/0.1% SDS at 25°C, and twice with 0.2× SSC/0.1% SDS at 60°C for 15 min, followed by 2 min with DIG washing buffer (0.5 M maleic acid, 0.75 M NaCl, 0.3% Tween 20, pH 7.5) at 25°C, 30 min with buffer 2 (0.5 M maleic acid, 0.75 M NaCl, 0.3% Tween 20, 20% blocking reagent) at 25°C, 30 min with buffer 2 and Anti-Digoxigenin-AP Fab fragments (1:10000) at 25°C, twice for 15 min with DIG washing buffer at 25°C and finally for 5 min in 100 mM Tris/100 mM NaCl/50 mM MgCl_2 _(pH 9.5) at 25°C. The membrane was swayed for 1 min in 1% CSPD solution (Roche) and subsequently exposed to Amersham Hyperfilm™ ECL (GE Healthcare).

### Amplification of 16S and 18S rRNA genes

Oligonucleotide primers targeting 16S rRNA or 18S rRNA gene signature regions were used for PCR to obtain near full-length bacterial 16S rRNA or amoeba 18S rRNA gene fragments of the novel isolates *Acanthamoeba *sp. WR (containing *A. asiaticus *WR) and *Acanthamoeba *sp. US1 (containing *A. asiaticus *US1); see Additional file 2, Table S1. Nucleotide sequences of DNA fragments were determined on an ABI 3130 XL genetic analyzer using the BigDye Terminator kit v3.1 (Applied Biosystems).

### Nucleotide sequence accession numbers

Obtained nucleotide sequences of IS elements of *A. asiaticus *EIDS3 and 16S and 18S rRNA genes of the isolates *Acanthamoeba *sp. WR (containing *A. asiaticus *WR) and *Acanthamoeba *sp. US1 (containing *A. asiaticus *US1) were submitted to EMBL/DDBJ/GenBank under accession numbers HM159367 to HM159370. The sequences of the *A. asiaticus *IS elements were deposited at EMBL/DDBJ/GenBank under accession numbers HM159371 to HM159380 and the ISFinder database http://www-is.biotoul.fr/[43].

## Authors' contributions

SSE and MH designed the study. SSE, TP and AS performed sequence analyses; TP and AS carried out the molecular biology experiments. SSE and MH wrote the manuscript; all authors read, edited, and approved the final manuscript.

## Supplementary Material

Additional file 1 **pdf-file containing Figures S1 to S12**. Click here for file

Additional file 2 **pdf-file containing Tables S1 to S32**. Click here for file
