# Supplementary material for: A bacterial genome in transition - an exceptional enrichment of IS elements but lack of evidence for recent transposition in the symbiont Amoebophilus asiaticus
Source: BMC Evol Biol. 2011 Sep 26;11:270. doi: 10.1186/1471-2148-11-270 (PMC3196728; doi:10.1186/1471-2148-11-270)
Supplement: Additional file 1 — pdf-file containing Figures S1 to S12. [file 1471-2148-11-270-S1.PDF]

Supplementary Figure S1

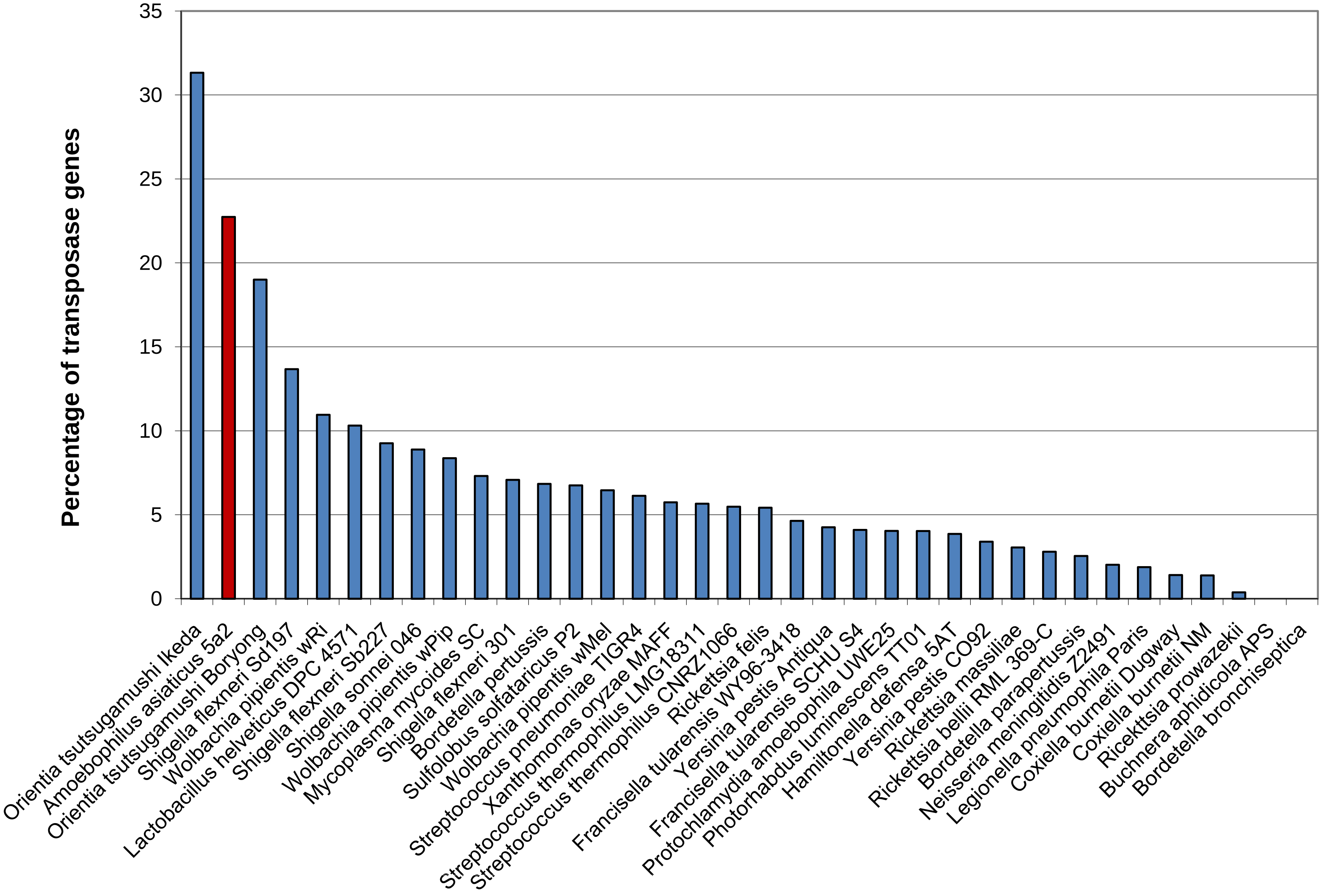

Supplementary Figure S1: Percentage of transposase genes in selected bacterial and archaeal genomes. The numbers of transposase genes in the respective genomes were taken from the publication describing the respective genome. Percentages of transposase genes were calculated based on genome sizes excluding plasmids. Only complete genomes were included in our analyses.

Supplementary Figure S2

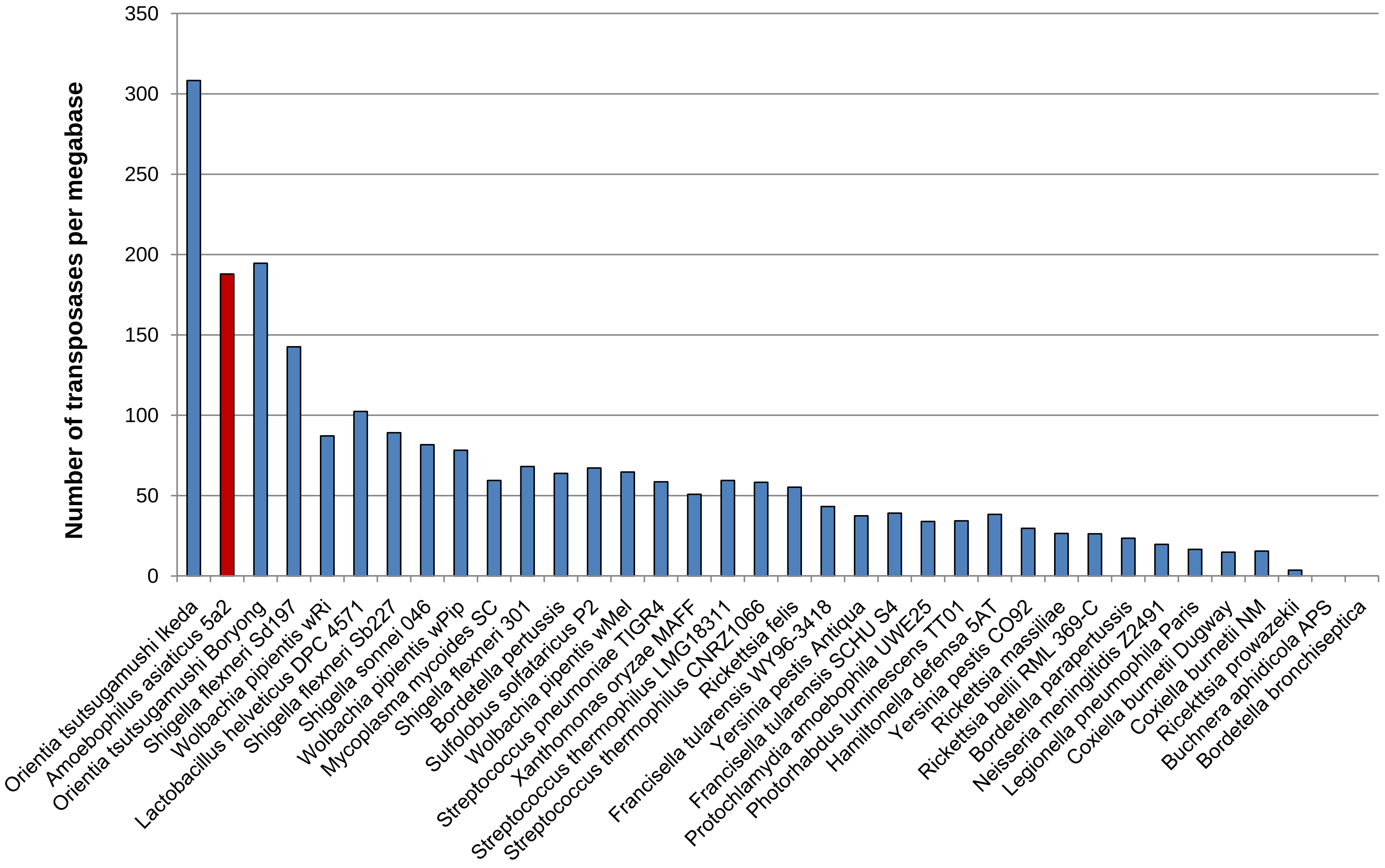

Supplementary Figure S2: Number of transposases per megabase in selected bacterial and archaeal genomes. The numbers of transposase genes in the respective genomes were taken from the publication describing the respective genome. The number of transposases per megabase genome was calculated based on genome sizes excluding plasmids. Only complete genomes were included in our analyses.

Supplementary Figure S3

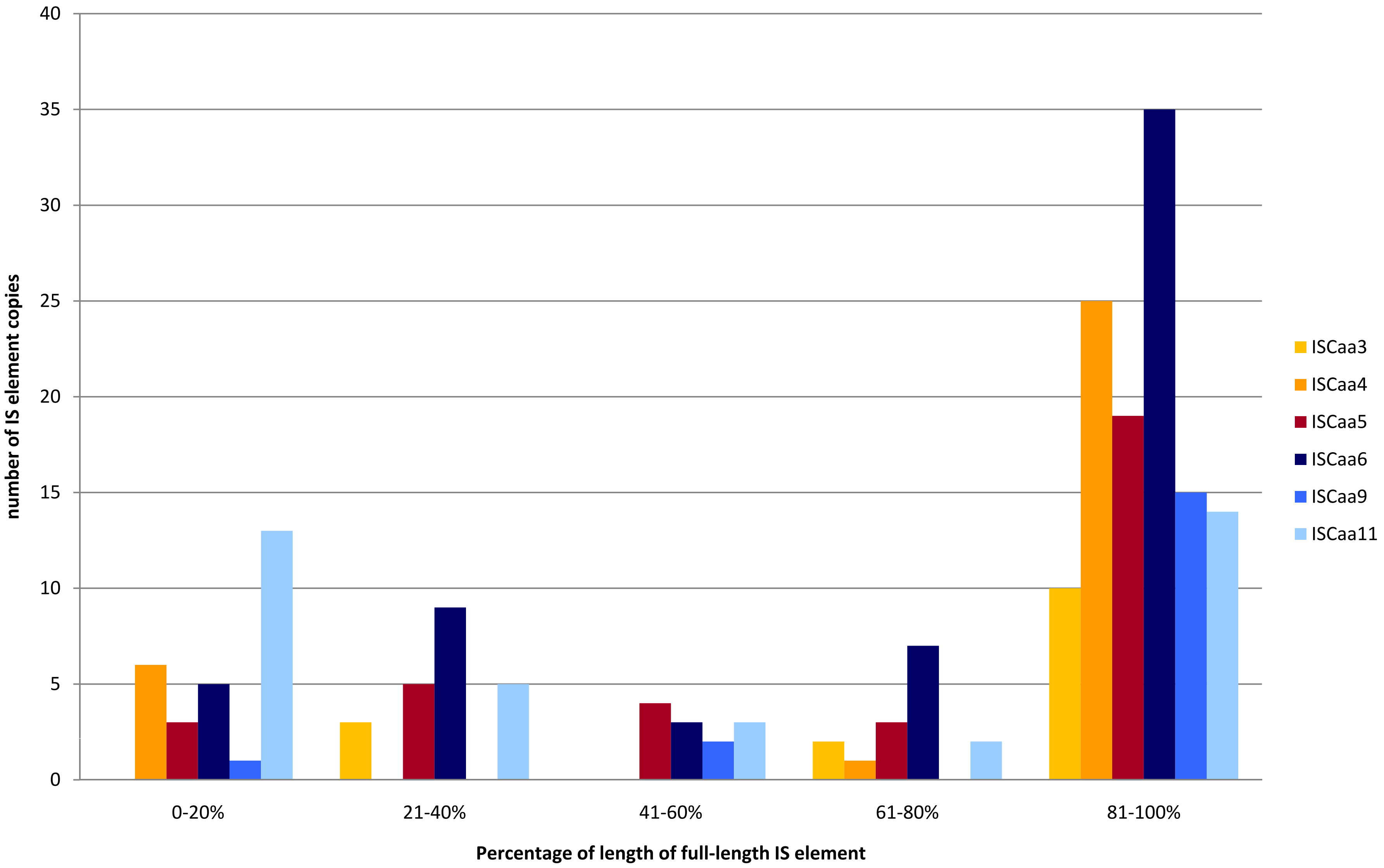

Supplementary Figure S3: Length distribution of IS element copies. The length distribution of full-length and partial IS element copies in the *A. asiaticus* genome is shown. Only IS elements which are present in at least 10 full-length copies are shown.

Supplementary Figure S4

```
1
                                 * V R G K S M N C P R C N N T Q S C K D G I V R G R Q R Y Q C K S C R
                                                  * I V L D V I I L K A V K M E L L E V D S A T S V K V A V
                                      * R K K Y E L S S M * * Y S K L * R W N C * R * T A L P V * K L P
GGTATTGTTAAATTGGGATAGGATTAGTAAGTTAGAGGAAAAAGTATGAATTGTCCTCGATGTAATAATACTCAAAGCTGTAAAGATGGAATTGTTAGAGGTAGACAGCGCTACCAGTGTAAAAGTTGCCG
---------IRL--------->

132
 F P Y T V S H K S D V K P V S T K R K A L Q L Y L E G L G F R A I G R I L N I S Y G T V
  S L T Q L V T N Q M L N L Y L L S E K R C N Y T * K D * D F E L * A V Y S T * A M E Q
F P L H S * S Q I R C * T C I Y * A K S V A I I L R R I R I S S Y R P Y T Q H K L W N S
TTTCCCTTACACAGTTAGTCACAAATCAGATGTTAAACCTGTATCTACTAAGCGAAAAGCGTTGCAATTATACTTAGAAGGATTAGGATTTCGAGCTATAGGCCGTATACTCAACATAAGCTATGGAACAG

263
 Y Q W V K A C G D Q V S L P E R Q D Q V D I V E M D E I H T Y V G S K K S T A G Y G *
S I N G * K H V E I K * V Y Q K D K I K * I * S R W M K Y T H M W V Q K S L L L D M D S
 L S M G K S M W R S S K F T R K T R S S R Y S R D G * N T H I C G F K K V Y C W I W I A
TCTATCAATGGGTAAAAGCATGTGGAGATCAAGTAAGTTTACCAGAAAGACAAGATCAAGTAGATATAGTCGAGATGGATGAAATACACACATATGTGGGTTCAAAAAAGTCTACTGCTGGATATGGATAG

394
L L I D * A S V L Y H M C V E I A R H K P D * S Y G S A S R I * A S C I V V I I G K A T
 C * * I E Q A F Y I I C V W R S L D T N R I E V M G A R Q G Y R Q A V L * * L L E K L P
 V D R L S K R F I S Y V C G D R S T Q T G L K L W E R V K D I G K L Y C S D Y W K S Y
CTGTTGATAGATTGAGCAAGCGTTTTATATCATATGTGTGTGGAGATCGCTCGACACAAACCGGATTGAAGTTATGGGAGCGCGTCAAGGATATAGGCAAGCTGTATTGTAGTGATTATTGGAAAAGCTAC

525
 S S L F Q K I N T D K A N Q K L I Q W K D I I A * * G T I * Q D L S V K E N A I A N R C
  A V Y S K R * T P T K Q I R N L Y S G R I * * L N E A L F S K I * A * R K M L * Q T G
Q Q F I P K D K H R Q S K S E T Y T V E G Y N S L M R H Y L A R F K R K G K C Y S K Q V
CAGCAGTTTATTCCAAAAGATAAACACCGACAAAGCAAATCAGAAACTTATACAGTGGAAGGATATAATAGCTTAATGAGGCACTATTTAGCAAGATTTAAGCGTAAAGGAAAATGCTATAGCAAACAGGT

656
  T * * K N R S T C * W P S * I I S C L S *
A H D R K I A Q P A N G Q A K *
 H M I E K S L N L L M A K L N N Q L P I L I *
GCACATGATAGAAAAATCGCTCAACCTGCTAATGGCCAAGCTAAATAATCAGCTGCCTATCTTAATTTAACAATACC
                                                     <---------IRR---------
```

Supplementary Figure S4: Sequence of ISCaa4. The DNA sequence and the translated amino acid sequences of ISCaa4 are shown. The inverted repeats are boxed and indicated ("IRL, IRR"). Start and stop codons are shown in boldface letters. The amino acid sequence of ORFA and ORFB are shown in blue and red, respectively. The slippery site for the predicted ribosomal -1 frameshifting between ORFA and ORFB (AAAAAAG) is shown in boldface letters and underlined. At the slippery site the reading frames are indicated by blue (ORFA) and red (ORFB) vertical bars. A 36 bp region five nucleotides downstream of the predicted slippery site for ribosomal frameshifting is predicted to form stem-loop structures (which have been described to stimulate ribosomal -1 frameshifting) is highlighted in grey. In the deduced amino acid sequences stop codons are indicated by asterisks. Nucleotide positions are indicated on top of each row.

Supplementary Figure S5

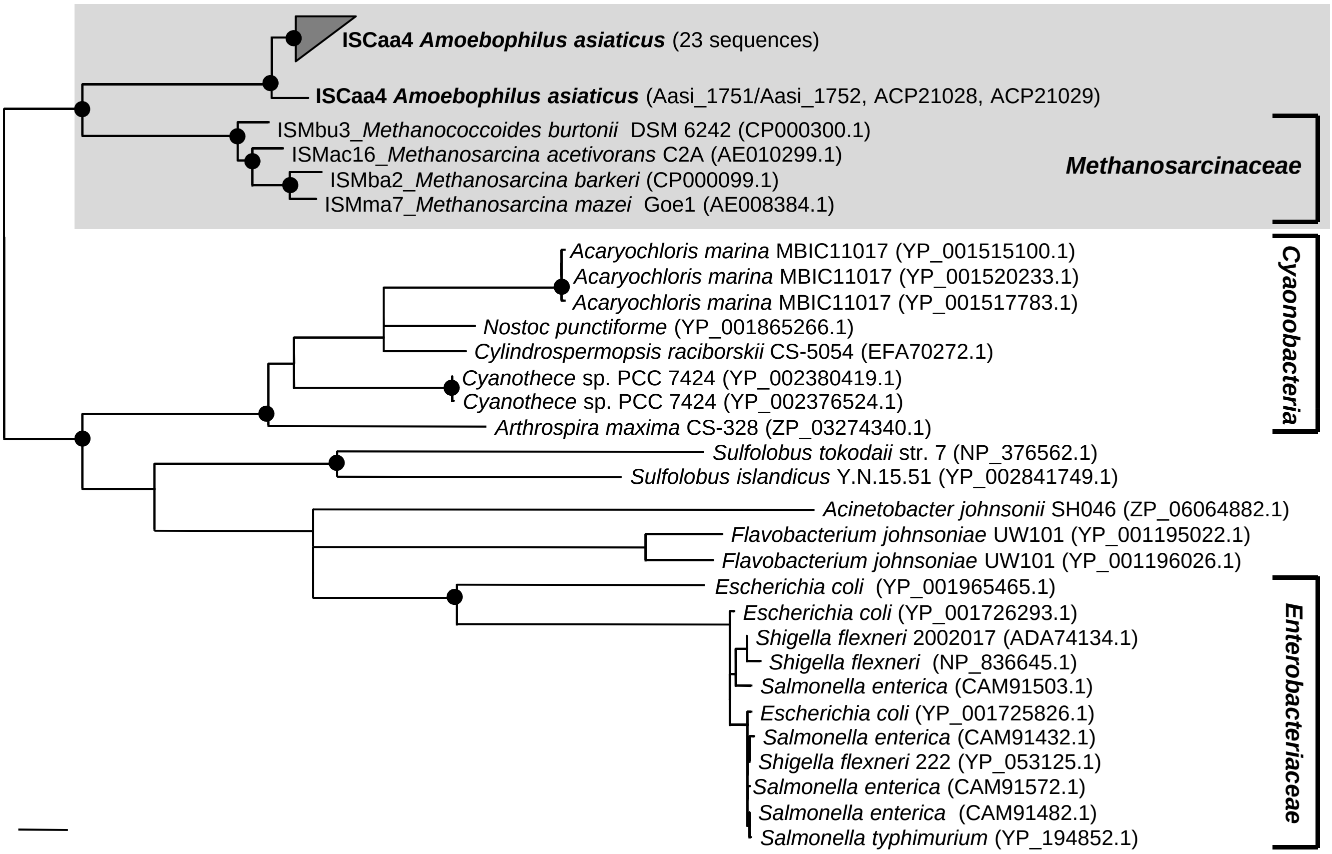

Supplementary Figure S5: Phylogenetic relationships of ISCaa4 and related IS1 family transposases.
An amino acid-based phylogenetic tree calculated with ARB using the TREE-PUZZLE algorithm is shown. Transposase sequences consisting of two ORFs were merged into a single ORF. Black dots indicate nodes which are supported by TREE-PUZZLE support values and maximum parsimony bootstrap values (1000x resampling) greater than 90%. GenBank accession numbers are indicated in brackets. The bar represents 10% estimated evolutionary distance.

Supplementary Figure S6

```
1
                                                  *  K  N  R  K  I  N  G  K  Q  S  S  K  N  L  K  A  R  R  I  P  S  L  N  R  R  K
                                       *  I  R  D  K  K  T  E  R  L  M  E  N  S  H  L  K  T  L  K  P  E  E  F  R  R  L  T  G  V  K
                               *  M  Y  K  L  G  I  K  K  Q  K  D  *  W  K  T  V  I  *  K  P  *  S  Q  K  N  S  V  A  *  Q  A  *  R
TAGAGTTCTTCTGAAACCTAAGGAATAAGCATAAATGTATAAATTAGGGATAAAAAAACAGAAAGATTAATGGAAAACAGTCATCTAAAAACCTTAAAGCCAGAAGAATTCCGTCGCTTAACAGGCGTAAA
---------IRL-------->
132
G  *  D  L  C  Q  N  V  S  Y  H  T  E  S  H  S  N  K  E  S  *  S  R  A  A  *  *  T  E  L  *  T  N  A  F  N  D  F  R  I  F  K  R  I
 A  E  T  F  A  K  M  L  A  I  I  Q  K  A  I  Q  T  K  K  A  K  V  G  R  P  N  K  L  S  C  E  Q  M  L  L  M  T  L  E  Y  L  R  E  Y
  L  R  P  L  P  K  C  *  L  S  Y  R  K  P  F  K  Q  R  K  L  K  *  G  G  L  I  N  *  V  V  N  K  C  F  *  *  L  *  N  I  *  E  N
GGCTGAGACCTTTGCCAAAATGTTAGCTATCATACAGAAAGCCATTCAAACAAAGAAAGCTAAAGTAGGGCGGCCTAATAAACTGAGTTGTGAACAAATGCTTTTAATGACTTTAGAATATTTAAGAGAAT

263
 P  H  I  L  P  Y  F  K  K  L  W  N  K  *  E  *  L  L  L  Y  H  P  F  Y  *  R  G  V  N  *  K  W  S  I  H  F  T  R  P  *  S  I  V  K
  R  T  Y  F  H  I  S  K  S  Y  G  I  S  E  S  N  C  Y  Y  T  I  R  F  I  E  E  A  L  I  K  S  G  Q  F  T  L  P  G  R  K  A  L  L
T  A  H  T  S  I  F  Q  K  V  M  E  *  V  R  V  I  A  I  I  P  S  V  L  L  K  R  R  *  L  K  V  V  N  S  L  Y  Q  A  V  K  H  C  *
ACCGCACATACTTCCATATTTCAAAAAGTTATGGAATAAGTGAGAGTAATTGCTATTATACCATCCGTTTTATTGAAGAGGCGTTAATTAAAAGTGGTCAATTCACTTTACCAGGCCGTAAAGCATTGTTA

394
 K  R  Y  G  I  C  S  N  L  N  R  C  Y  R  E  P  H  *  A  A  *| K| K  Q  R  Y  Y  Y  S  G  K  K  K  R  H  T  L  K  T  Q  L  V  V
K  S  D  M  E  Y  A  V  I  L  I  D  A  T  E  S  P  I  E  R  P  K  K| S  S  D  I  T  T  Q  V  K  R  N  D  I  H  *  R  H  N  *  L  S
 K  A  I  W  N  M  Q  *  S  *  *  M  L  Q  R  A  P  L  S  G  L  K  K  A  A  I  L  L  L  R  *  K  E  T  T  Y  T  K  D  T  T  S  C  R
AAAAGCGATATGGAATATGCAGTAATCTTAATAGATGCTACAGAGAGCCCCATTGAGCGGCCTAAAAAAAGCAGCGATATTACTACTCAGGTAAAAAGAAACGACATACACTAAAGACACAACTAGTTGTC

525
D  K  Q  T  R  A  I  I  C  S  S  F  S  N  G  K  R  H  D  F  R  L  F  K  E  S  K  V  H  I  S  Q  H  S  Q  A  I  V  D  T  G  Y  Q  G
 I  S  K  R  E  P  *  Y  V  V  V  F  Q  M  E  S  G  M  T  F  A  C  L  R  N  L  R  Y  I  *  A  S  I  V  K  P  *  *  I  Q  A  I  K  G
  *  A  N  A  S  H  N  M  *  *  F  F  K  W  K  A  A  *  L  S  L  V  *  G  I  *  G  T  Y  K  P  A  *  S  S  H  S  R  Y  R  L  S  R
GATAAGCAAACGCGAGCCATAATATGTAGTAGTTTTTCAAATGGAAAGCGGCATGACTTTCGCTTGTTTAAGGAATCTAAGGTACATATAAGCCAGCATAGTCAAGCCATAGTAGATACAGGCTATCAAGG

656
 L  K  R  L  H  D  K  S  L  M  P  K  K  R  S  K  K  H  P  L  T  K  A  D  K  R  S  N  Q  Q  L  A  S  Q  R  V  L  S  E  H  I  I  G  M
  *  R  G  Y  M  T  N  R  L  C  L  K  N  A  V  K  S  I  L  *  P  K  P  I  R  G  V  I  S  N  *  P  A  K  G  C  L  A  S  I  *  L  V
V  K  E  A  T  *  Q  I  A  Y  A  *  K  T  Q  *  K  A  S  F  D  Q  S  R  *  E  E  *  S  A  I  S  Q  P  K  G  A  *  R  A  Y  N  W  Y
GTTAAAGAGGCTACATGACAAATCGCTTATGCCTAAAAAACGCAGTAAAAAGCATCCTTTGACCAAAGCCGATAAGAGGAGTAATCAGCAATTAGCCAGCCAAAGGGTGCTTAGCGAGCATATAATTGGTA

787
 L  K  R  F  K  I  L  A  E  R  Y  R  N  R  R  K  R  F  G  L  R  F  N  L  I  A  G  I  Y  N  Y  E  L  L  C  *
C  S  S  G  L  K  F  *  R  S  V  T  E  I  G  E  K  D  L  G  Y  A  L  I  L  L  L  E  F  I  T  M  N  F  Y  V  K  F  R  K  N  S
 A  Q  A  V  *  N  F  S  G  A  L  Q  K  *  E  K  K  I  W  A  T  L  *  S  Y  C  W  N  L  *  L  *
TGCTCAAGCGGTTTAAAATTTTAGCGGAGCGTTACAGAAATAGGAGAAAAAGATTTGGGCTACGCTTTAATCTTATTGCTGGAATTTATAACTATGAACTTTTATGTTAAGTTTCGGAAGAACTCTA
                                                                                                              <--------IRR---------
```

Supplementary Figure S6: Sequence of ISCaa3. The DNA sequence and the translated amino acid sequences of ISCaa3 are shown. The inverted repeats are boxed and indicated (“IRL, IRR”). Start and stop codons are shown in boldface letters. The amino acid sequence of ORFA and ORFB are shown in blue and red, respectively. The slippery site for the predicted ribosomal -1 frameshifting between ORFA and ORFB (AAAAAAG) is shown in boldface letters and underlined. At the slippery site the reading frames are indicated by blue (ORFA) and red (ORFB) vertical bars. In the deduced amino acid sequences stop codons are indicated by asterisks. Nucleotide positions are indicated on top of each row.

Supplementary Figure S7

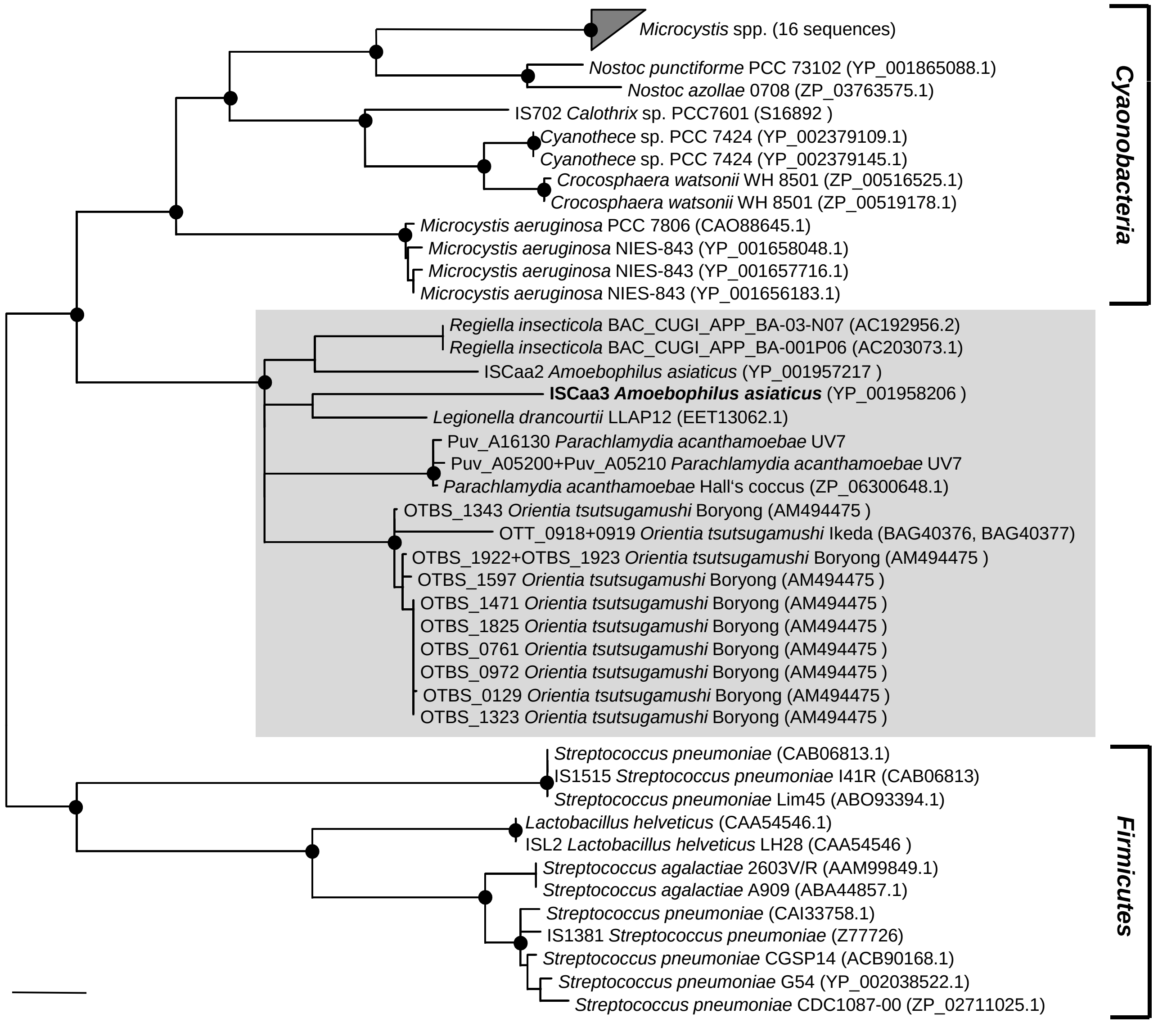

Supplementary Figure S7: Phylogenetic relationships of ISCaa3 and related IS5 family transposases.

An amino acid-based phylogenetic tree calculated with ARB using the TREE-PUZZLE algorithm is shown. Transposase sequences consisting of two ORFs were merged into a single ORF. Black dots indicate nodes which are supported by TREE-PUZZLE support values and maximum parsimony bootstrap values (1000x resampling) greater than 90%. GenBank accession numbers are indicated in brackets where available, otherwise locus_tags are given. The bar represents 10% estimated evolutionary distance. The sequences of the IS elements from *Parachlamydia acanthamoebae* sp. UV7 were taken from the genome of *P. acanthamoebae* sp. UV7 (Collingro at al.: "Unity in Variety – the Pan-Genome of the *Chlamydiae"*, Mol. Biol. Evol., *in press*).

Supplementary Figure S8

```
1
                                                                    * T N S Y A I H * R F N R * G M G S V
                                                          * L V N F K L T A M P Y T S D L T D K E W E V L
                                                                           * Q L C H T L A I * Q I R N G K C
TCTAAGAGGCTGTCTAAAAAGGGTAATTTAGATAGGAAAAGCAGACAAAACTTTATAAAATTAGTTAGTGAACTTTAAACTAACAGCTATGCCATACACTAGCGATTTAACAGATAAGGAATGGGAAGTGT
--------IRL--------->

132
R T I S Y L H R S L P T P * I H Y K R S I R C Y F L F R K N R L S M A H A T C P F S T L
 E P L V T Y I G P C R P R K Y T I R E V L D A I F Y L E K T G C Q W R M L P A H F P P
* N H * L L T * V L A D P V N T L * E K Y * M L F S I * K K Q A V N G A C Y L P I F H P
TAGAACCATTAGTTACTTACATAGGTCCTTGCCGACCCCGTAAATACACTATAAGAGAAGTATTAGATGCTATTTTCTATTTAGAAAAAACAGGCTGTCAATGGCGCATGCTACCTGCCCATTTTCCACCC

263
 K K C L * L V L S L A K K W * M G L S P * Y T S R S S T R K V G Q T S H T * R C H Y G
W K S V Y D L Y Y R W R R N G K W D L V H D T L R D Q V R E K L G K Q A I P S A A I M D
 E K V F M T C I I A G E E M V N G T * S M I H F E I K Y E K S W A N K P Y L A L P L W I
TGAAAAAGTGTTTATGACTTGTATTATCGCTGGCGAAGAAATGGTAAATGGGACTTAGTCCATGATACACTTCGAGATCAAGTACGAGAAAAGTTGGGCAAACAAGCCATACCTAGCGCTGCCATTATGGA

394
* P L G * N S T K R G S R G Y D A A K|K|V K G R K Q H I I V D T L G L I L C V V V H S A
 S R S V K T A Q K G G R E A M M Q Q|K|K|* K A V S S I L L L I R W G L Y F V W * Y I Q L
 A A R L K Q H K K G V E R L * C S K|K|S K R P * A A Y Y C * Y A G A Y T L C G S T F S
TAGCCGCTCGGTTAAAACAGCACAAAAAGGGGGTCGAGAGGCTATGATGCAGCAAAAAAAGTAAAAGGCCGTAAGCAGCATATTATTGTTGATACGCTGGGGCTTATACTTTGTGTGGTAGTACATTCAGC

525
H L S D S R G C H L L L I R L F N T F P S I K H I W V D G G Y Q K G C I M W A Y G V L G
 T Y Q T Q E D V I F C L F A F L I H S L L L N T Y G * M E D T R K D V * C G L M G C *
S L I R L K R M S S F A Y S P F * Y I P F Y * T H M G R W R I P E R M Y N V G L W G A R
TCACTTATCAGACTCAAGAGGATGTCATCTTTTGCTTATTCGCCTTTTTAATACATTCCCTTCTATTAAACACATATGGGTAGATGGAGGATACCAGAAAGGATGTATAATGTGGGCTTATGGGGTGCTAG

656
 Y T L E V V Q R L S N G F H I L K K R W I V E R T F A W L S F S R R L S K D Y E H N P
V I L * K L Y N G S Q M G F I F * K S D G * S S V H L L G * V F L G G S L K I T S I I P
 L Y F R S C T T A L K W V S Y S K K A M D S R A Y I C L A E F F * E A L * R L R A * S Q
GTTATACTTTAGAAGTTGTACAACGGCTCTCAAATGGGTTTCATATTCTAAAAAAGCGATGGATAGTCGAGCGTACATTTGCTTGGCTGAGTTTTTCTAGGAGGCTCTCTAAAGATTACGAGCATAATCCC

787
K S S E T H I K I V M I R I M L K R L L *
 S L L K H I L K L L *
  V F * N T Y * N C Y D * N H A *
AAGTCTTCTGAAACACATATTAAAATTGTTATGATTAGAATCATGCTTAAGCGGCTTTTATGACTCCATTTTGGCTTTTTAGACAGCCTCTTAGA
                                                                          <-------IRR----------
```

Supplementary Figure S8: Sequence of ISCaa9. The DNA sequence and the translated amino acid sequences of ISCaa9 are shown. The inverted repeats are boxed and indicated ("IRL, IRR"). Start and stop codons are shown in boldface letters. The amino acid sequence of ORFA, ORFB and ORFC are shown in blue, purple and red, respectively. The proposed stop codon read-through (UGA, bp 263-265) is shown in boldface and underlined. The slippery site for the predicted ribosomal -1 frameshifting between ORFB and ORFC (AAAAAAG) is also shown in boldface letters and underlined. At the slippery site the reading frames are indicated by purple (ORFB) and red (ORFC) vertical bars. A 40 bp region five nucleotides downstream of the predicted slippery site for ribosomal frameshifting is predicted to form stem-loop structures (which have been described to stimulate ribosomal -1 frameshifting) is highlighted in grey. In the deduced amino acid sequences stop codons are indicated by asterisks. Nucleotide positions are indicated on top of each row.

Supplementary Figure S9

```
ISMac15            1 ---------------------MSEQTERLPYPSDLSDKEWKLIEPHIPNPPTNRGKKRVH
ISR12              1 VILFVCLGRTETTMGWTDFTRRQYARRTVRYASDLTDREWGLISPCLP-GPRRLGRPRST
IS1032             1 -------------MAWTEITRAQYRRDDLEYASDLRDAEWALIAPLMP-EKKRLGRPRRT
ISCaa9_5a2         1 ----------------------------MPYTSDLTDKEWEVLEPLVT--YIGPCRPRKY
ISCaa9_EIDS3       1 ------------------------------------------------------------
ISWpi1             1 --------------------------MRKKYPTDLSEREWARIEKHFRVSYKKGGRPPKY
consensus          1              ..... .. .. ...... ............. ..   ........

                                                  ▼
ISMac15           40 PYREILNGIFYLLRSGCAWRMLPHEFPPWQTVYHYFRLWRLYGIWERINAALRTELRIAN
ISR12             60 DLREVVNALLYIATTGCQWRMMPRDFPPFTTVQSYFYEWRATGLWGRINHHLVMEARDLE
IS1032            47 DLRRVMEAILYIVTTGCQWRQLPRHFPAFTTVQGYFYRWIREGRWEAINHILVILSREQD
ISCaa9_5a2        31 TIREVLDAIFYLEKTGCQWRMLPAHFPPWKSVYDLYYRWRRNGKWDLVHDTLRDQVREKL
ISCaa9_EIDS3       1 --------------------MLPAHFPPWKSVYDLYYRWRKNEKWDLVHDTLRDQVREKL
ISWpi1            35 SKREILEAIFYVLRTGCQWRYLPNDFPLWKTVYEQFRQWKKQGIFEKMNYEITKYSRRKI
consensus         61 ......................*..**....*......*.... ..... .... .*...

                                N2 (71 amino acid residues)
                                ▼
ISMac15          100 GREPEPSAAILDSQSVKTTETRGVRGYDAGKKVKRRKRHILVDTTGLILMVVVHAANIQD
ISR12            120 GREASPSAGVIDSQSVKTTESGGISGYDAGKKIKGRKRHIVVDTLGLMVGLMVHSADIQD
IS1032           107 GRDATPSDGIIDSQSVKTAENGGPRGYDAGKKIRGRKRHITTDTLGHVVAAVVHPADIQD
ISCaa9_5a2        91 GKQAIPSAAIMDSRSVKTAQKGGREAMMQQKKVKGRKQHIIVDTLGLILCVVVHSAHLSD
ISCaa9_EIDS3      41 GKRAIPSAAIMDSRSVKTAQKGGQKAMMQQKKVKGRKQHIVVDTLGLILCVVVHSAHLSD
ISWpi1            95 GRNEQPSACIVDSQSVKTTEKGGSKAMMEVKKVKGRKRHIITDTQGFILGCYVGAANEND
consensus        121 *....**....**.**** ...*...  ..**...**.**..**.*......*..* ..*

                                            N3 (38 amino acid residues)
                                            ▼
ISMac15          160 RDGAKLVLEQIKGTFSRLQLIWADAAYAGQ-LVDWVKITCGWVLEIVRR-----------
ISR12            180 RDGAPAVLKTILKRWPWLRHIFADGGYAGSKLRGALQKIAAFTLQIVKR-----------
IS1032           167 RDGAPLVATRIRSLFPWLRHLIGDGGYAGEKLRGALAELGRWTIEIVKR-----------
ISCaa9_5a2       151 SRGCHLLLIRLFNTFPSIKHIWVDGGYQKGCIM-WAYGVLGYTLEVVQR-----------
ISCaa9_EIDS3     101 SRGCHLLLIRLFNAFPSIKHIWVDGGYQKGCII-WAYGVLDYTLEVVKR-----------
ISWpi1           155 RDGIKIALNNMRTKYTKVKKMWADMGYQGRNLKNHIKEEYDIDIEIVKRPPCRFWVHKDT
consensus        181 ..*............. ......*..* .. .....  ........*.*

                                         C1
                                          ▼
ISMac15          208 -----KDDVKGFQVLPRRWVVERTFGWLGRYRRLSKDYEGLTESSQAFIYAAMIHIMSRR
ISR12            229 -----TDKAKGFEVLPRRWVVERTFAWLGRCRRLAKDWEKSVASAEAWVTIAHIRVLTRR
IS1032           216 -----SDRAEGFVVLPKRWIVERSFAWLGRCRRLTKDVEATISSSHAWLMIAHIRRVLRK
ISCaa9_5a2       199 -------LSNGFHILKKRWIVERTFAWLSFSRRLSKDYEHNPKSSETHIKIVMIRIMLKR
ISCaa9_EIDS3     149 -------ISNGFSILKKRWIVERTFAWLSFSRRLSKDYEHNPKSSETHIKIVMIRIMLKR
ISWpi1           215 PPELLPTREQGFKVQPRRWVVERTFAWVNRNRRLSKEYDLLTTSTENFIYLAMSRVMLKR
consensus        241      ... .**.....**.***.*.*...  ***.*....  .*..... ..........

ISMac15          263 LAKIEPLSR
ISR12            284 LARYG--YR
IS1032           271 INQTA--F-
ISCaa9_5a2       252 L-------L
ISCaa9_EIDS3     202 L--------
ISWpi1           275 E------YA
consensus        301 ... .  ..
```

Supplementary Figure S9: Alignment of amino acid sequences of ISCaa9 and selected homologous IS5 family transposase sequences. The alignment was done with MAFFT (Katoh et al., 2008, Brief. Bioinform. 9:286-98), shading of conserved amino acid residues was performed with Boxshade available at the Swiss EMBnet server (http://www.ch.embnet.org/index.html). The N2, N3 and C1 domains of DDE-motif transposases are indicated and highlighted in boldface letters, the distance between these residues are indicted in brackets. Conserved amino acid residues of IS5 family IS1031 group transposases are shown in red, (Mahillon and Chandler, 1998, Microbiol. Mol. Biol. Rev. 62(3):725-74). The position of the predicted stop codon read-through and proposed recoding into tryptophane in ISCaa9 (from *A. asiaticus* 5a2) is indicated by a green arrowhead and green letters. Interestingly, the ISCaa9-like IS element from *A. asiaticus* EIDS3 is predicted to consist of only two ORFs, the proposed stop codon read-through between ORFA and ORFB in ISCaa9 from *A. asiaticus* 5a2 is not conserved (TGG (Trp) instead of TGA). A consensus line is displayed at the bottom of each alignment block, an asterisk indicates identical positions, a dot indicates similar positions. Abbreviations and accession numbers: ISMac15: ISMac15 *Methanosarcina acetivorans* C2a, NC_003552; ISR12: ISR12 *Rhizobium leguminosarum* biovar viciae MSDJ4184, Z37965; IS1032: IS1032 *Acetobacter xylinum*, M80805; ISCaa9_5a2: ISCaa9 *Amoebophilus asiaticus* 5a2; ISCaa9_EIDS3: ISCaa9 *Amoebophilus asiaticus* EIDS3, HM159371; ISWpi1: ISWpi1 *Wolbachia pipientis* wPip, NC_002978, Cordaux, 2008, Gene 409(1-2):20-7.

Supplementary Figure S10

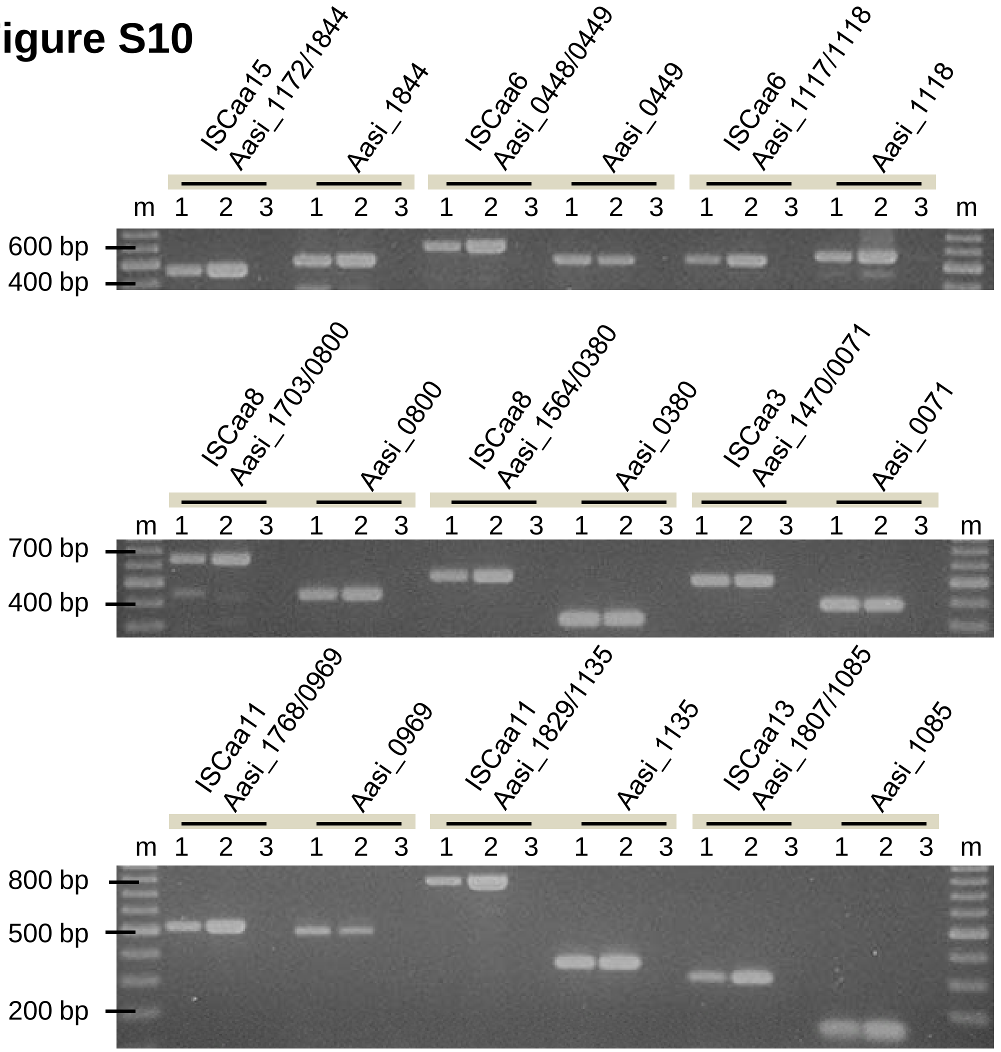

Supplementary Figure S10: Comparision of contiguous transcription of IS elements and their downstream genes with the transcription of downstream genes alone during intracellular growth of *A. asiaticus* 5a2 in its *Acanthamoeba* host. Transcription of IS elements was analyzed with reverse transcriptase PCR. Lanes 1: PCR with cDNA ; lanes 2: PCR with genomic DNA as positive control; lanes 3: PCR without nucleic acid as negative control. See Figure 4 for details on the genomic organization of the analyzed loci. Details for the properties of the downstream genes can be found in Supplementary Table 3.

Supplementary Figure S11:

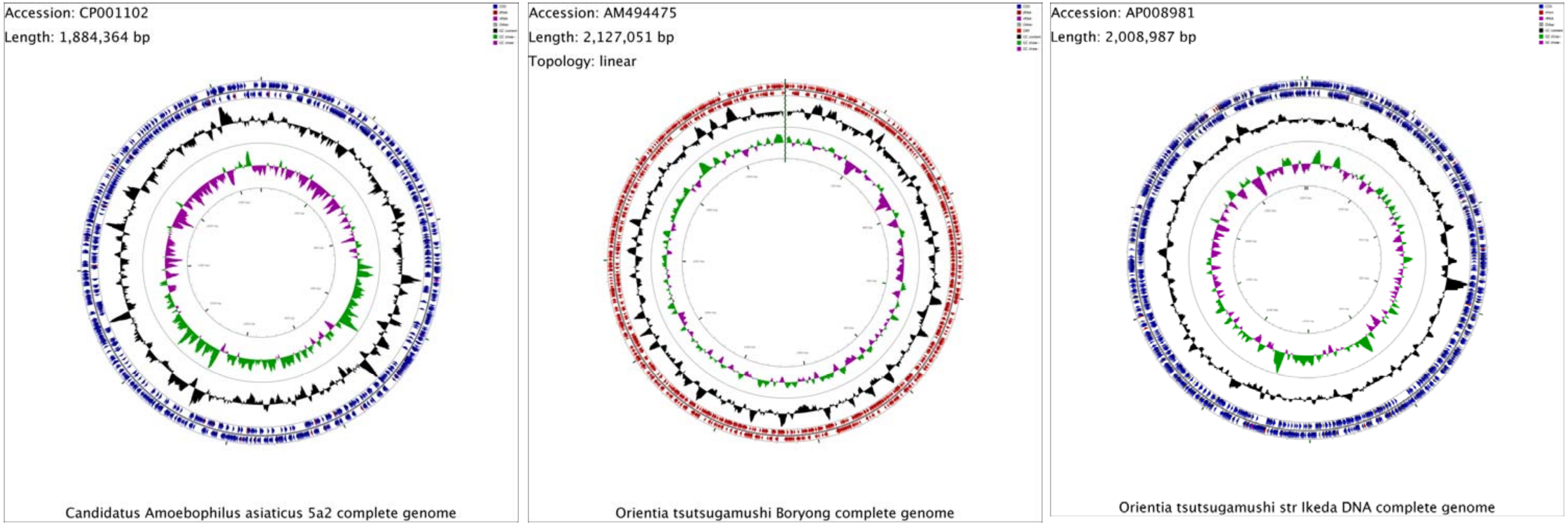

Supplementary Figure S11: Visualisation of the GC-skew pattern in selected prokaryotic genomes. The genomes of selected prokaryotes containing high numbers of IS elements (see also Supplementary Figures S1 and S2 for details) are shown. The visualization of genomes and GC skew patterns was done with the CGView webserver http://stothard.afns.ualberta.ca/cgview_server/, (Grant JR, Stothard P, 2008, Nucleic Acids Res. 2008;36 (Web Server issue):W181-4). The outer two circles represent predicted CDSs on the plus and minus strand, respectively; third circle (black): GC content; fourth circle (green, pink): GC-skew. GenBank accession numbers are indicated on top of each panel.

Accession: NC_007606

Length: 4,369,232 bp

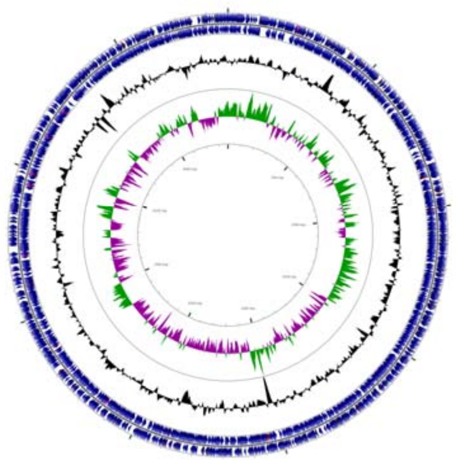

Shigella dysenteriae Sd197 complete genome

Accession: CP000036

Length: 4,519,823 bp

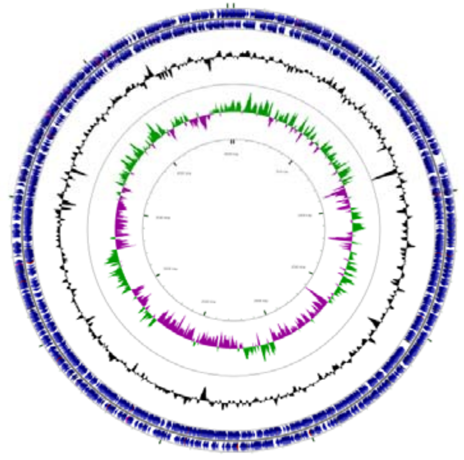

Shigella boydii Sb227 complete genome

Accession: CP000038

Length: 4,825,265 bp

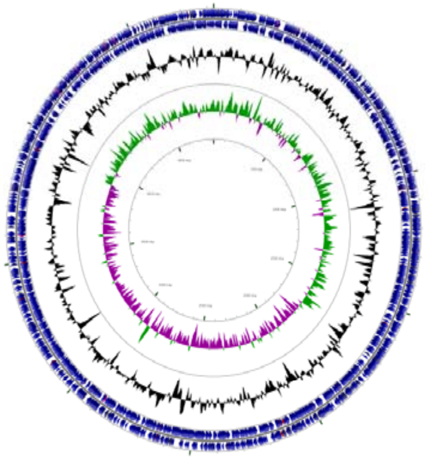

Shigella sonnei Ss046 complete genome

Accession: AE017196

Length: 1,267,782 bp

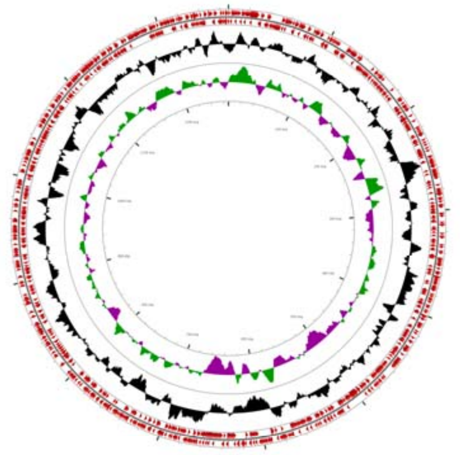

Wolbachia endosymbiont of Drosophila melanogaster complete genome

Accession: AM999887

Length: 1,482,455 bp

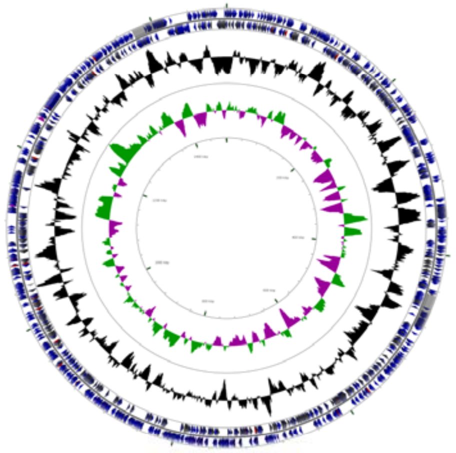

Wolbachia sp. wPip complete genome

Accession: CP001391

Length: 1,445,873 bp

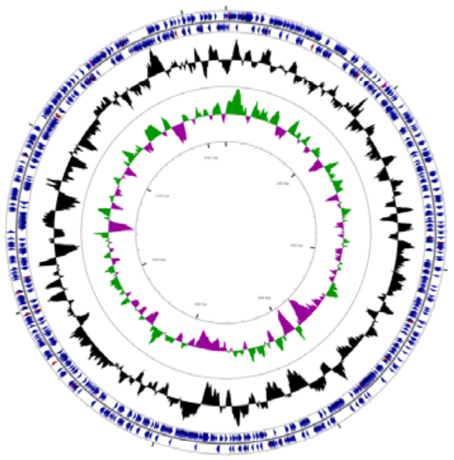

Wolbachia sp wRi complete genome

Accession: NC_010080

Length: 2,080,931 bp

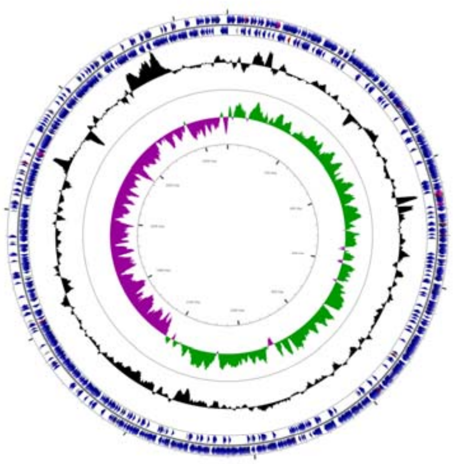

Lactobacillus helveticus DPC 4571 complete genome

Accession: NC_002929

Length: 4,086,189 bp

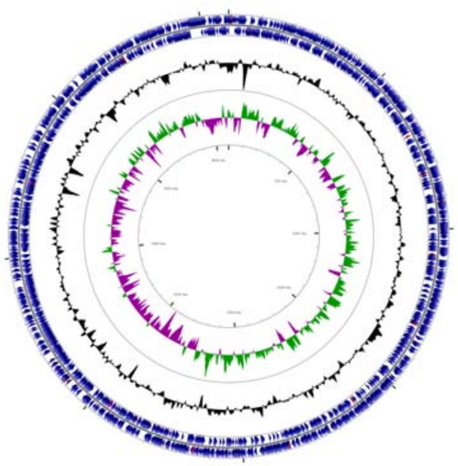

Bordetella pertussis Tohama I complete genome

Accession: NC_002754

Length: 2,992,245 bp

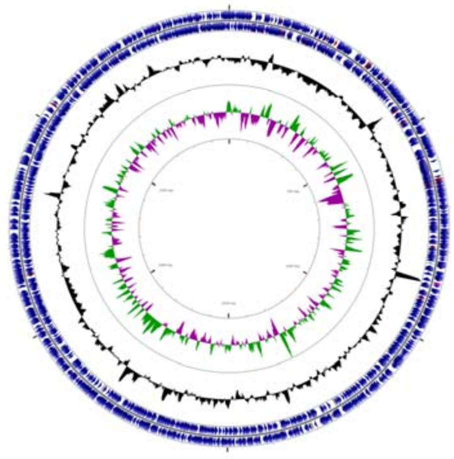

Sulfolobus solfataricus P2 complete genome

Accession: NC_005364

Length: 1,211,703 bp

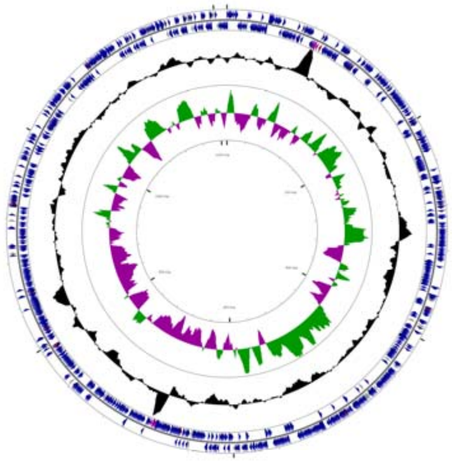

Mycoplasma mycoides subsp mycoides SC str PG1 complete genome

Supplementary Figure S12:

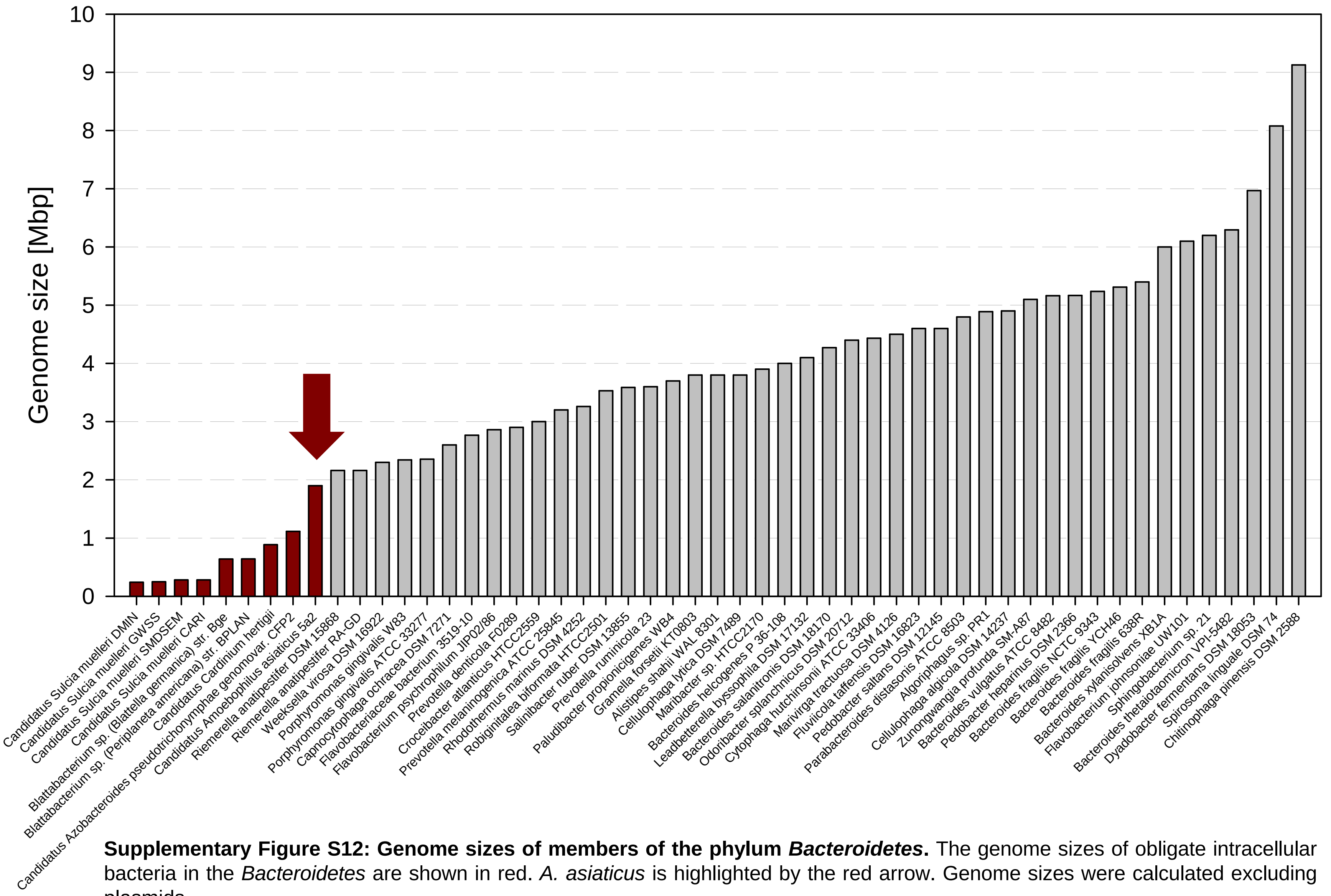

Supplementary Figure S12: Genome sizes of members of the phylum *Bacteroidetes*. The genome sizes of obligate intracellular bacteria in the *Bacteroidetes* are shown in red. *A. asiaticus* is highlighted by the red arrow. Genome sizes were calculated excluding plasmids.
