# Supplementary material for: A bacterial genome in transition - an exceptional enrichment of IS elements but lack of evidence for recent transposition in the symbiont Amoebophilus asiaticus
Source: BMC Evol Biol. 2011 Sep 26;11:270. doi: 10.1186/1471-2148-11-270 (PMC3196728; doi:10.1186/1471-2148-11-270)
Supplement: Additional file 2 — pdf-file containing Tables S1 to S32. [file 1471-2148-11-270-S2.PDF]

Supplementary Table S1: Primers used for amplification of IS elements, loci with IS elements and their downstream genes, amplification of 16S and 18S rRNA genes and generation of probes for southern hybridizations.

IS element, genomic locus	Forward primer sequence (5‘ - 3‘)	Reverse primer sequence (5‘ - 3‘)	Expected length of amplificate [bp]	Annealing temperature [°C]
ISCaa2[a]	TTG GGT GTA AAA AGA GGC AAT	GCA TGG CGA TAA CAT TTT CA	745	63.7
ISCaa3[a]	AAC AGG CGT AAA GGC TGA GA	AAC CGC TTG AGC ATA CCA AT	680	63.7
ISCaa4[a]	AGA GGT AGA CAG CGC TAC CAG	TAG CTT GGC CAT TAG CAG GT	602	63.7
ISCaa5[a]	GCC TTA TAC TTT GGC GGA CA	TGC ACT ACC AGC AGG CAT AG	423	63.7
ISCaa6[a]	GCC GCT TGC TAA GAA AAA TG	AAA GAA GGG AGC TTG GCT CT	600	63.7
ISCaa7[a]	AAA GCT CGC CAA TTC TCA AC	TTG GCC ATC GGA TGT ACT TT	443	63.7
ISCaa8[a]	CAG CCC GCA AGA AAA CTA AA	CAG CCA AAG CTC CGT TCT AC	620	63.7
ISCaa9[a]	CAT GCT ACC TGC CCA TTT TC	TAA AAG CCG CTT AAG CAT GA	609	63.7
ISCaa10[a]	CTT GCG GCG TAA ATT GCA TA	CAG CGG GGT ATT CAA AAC AA	476	63.7
ISCaa11[a]	TAC ATA AAT GCG CCA GGA CA	TGC CTT CTT GCT CAC AAA TG	660	63.7
ISCaa12[a]	TTC AAG AAG CCC ATG GAG AG	GCA TAG GCG TTT TCC CAT AA	534	63.7
ISCaa15[a]	AGC ATG GCT TGT TGG AGA GT	CGC TCT TGA TCC GTA AGC TC	419	63.7
ISCaa16[a]	GGG CTA TAG CTG GCG TGT AA	GCA TTA AAA CGC CCC TTC TT	432	66.4
Aasi_1470/0071 (ISCaa3)	AAG GGT GCT TAG CGA GCA TA	CGC ATT CTG AAA CGC TAT GA	496	63.7
Aasi_0448/0449 (ISCaa6)	ATC AAG GCT GGC AAA AGC TA	CTG CCC GAA TGC CTA TAA TC	575	45.0
Aasi_1117/1118 (ISCaa6)	ATC AAG GCT GGC AAA AGC TA	TTA TCC CCC AAC CAT TTT CA	503	45.0
Aasi_1703/0800 (ISCaa8)	CGG TTG TGG AAG AAC TGT GA	AGT CCC CAT GCT TTC AGA TG	633	46.7
Aasi_1564/0380 (ISCaa8)	AAT GGT GGT GCT TGC TTT TC	CTC TTC TTC AGC CTG CTC GT	519	53.7
Aasi_1768/0969 (ISCaa11)	CAA GCT GTG TCG GCA AGT AA	ATC CGA TCT GTA GCG TTT GG	504	68.0
Aasi_1829/1135 (ISCaa11)	CAA GCT GTG TCG GCA AGT AA	AAA AGT GCT GTG GTC CAA CC	769	68.0
Aasi_1807/1085 (ISCaa13)	TTT GAT CGA GCA TTT CTT TGC	AAG GGA GTA TGT CCT GCA TCA	300	49.6
Aasi_1172/1844 (ISCaa15)	TGC AAG AAC CAA CAT TCA TTG	GAA GAA ATA GGG GAG CAC CA	454	59.5
Aasi_0897/1745 (ISCaa16)	AGA ATC AGA CCA TGG CAA GC	AGC CGG GTC TTT ATG AGG AT	487	66.4
Aasi_1844	ATT GGC TGG GGA CTC TTT TT	TTC GTG GTC TGG GTG TTG TA	490	59.6
Aasi_0449	GTG GAG TTC TAG CGG ACT GC	AGA GTT TCT GCC AGC TCT GC	510	53.7
Aasi_1118	TGC TGA AAG GGG AAG TTC AG	CGC AGC GGC TCT ATT GTA TT	541	53.7
Aasi_0800	TGA TCG GAA AAT GGA TGG AT	CAT TAG CTG CCT CCT GGG TA	428	63.7
Aasi_0380	AGC TAT GGC TTC TTG GTG TGA	GCT CAT TAA CGA ACC ACT GC	305	49.6
Aasi_0071	CGC CAG CAG TAA TTA ATG GAA	GCA ACT GGT AGA CCG GAA AA	374	63.7
Aasi_0969	CAT GCC AAA AGA ACG CCT AT	TGT TTC CGC ATG TTG TTC AT	487	68.0
Aasi_1135	GGT TGG ACC ACA GCA CTT TT	TGG CAC AAA TAT AAA ACC TGG A	348	59.6
Aasi_1085	TGG TTG AGG CTC TTT TAG CA	CCA CCC TGC TTA CTT AAT AGT TCA	130	49.6
Aasi_1200/1201	CAC AGG GAT TCG GTG TTC TT	AAG AGC GTG GTG CTT TGA AT	784	66.4
Aasi_0308/0309	GGA AAT ACG GAG GCA CAG AA	CCT GCT GCC ACG TTT TTA AT	471	59.6
**16S rDNA:** Aas79F/1281R	ACA CTT CGG TGT TGC TGG	ATT GGC CGC TTG TTA CAA	1203	50.0
**16S rDNA:** Aas79F/1467R[b]	ACA CTT CGG TGT TGC TGG	GTC GCT GAT CTA ACC CTA	1405	50.9
**18S rDNA:** 18SF/18SR[b]	GTA GTC ATA TGC TTG TCT C	CGR ARA CCT TGT TAC GAC	~ 2200	61.1

[a] these primers were also used for generation of probes for southern hybridizations

[b] Schmitz-Esser et al., 2008, Appl Environ Microbiol. Sep;74(18):5822-31.

Supplementary Table S2: Calculations for the sensitivity of Southern hybridizations

Copy number of IS element in the *A. asiaticus* genome	Detection limit (DL)	Detected IS element copies (C)	Detected *A. asiaticus* cells (N)	Detected number of infected amoeba subpopulation cells (DS)
1	1	92 646	92 646	93
	2	27 793	27 793	28
7	1	92 646	13 235	13
	2	27 793	3 970	4
24	1	92 646	3 860	4
	2	27 793	1 158	1

Sensitivity of Southern hybridizations was calculated assuming different copy numbers of the IS elements in the *A. asiaticus* genome (1, 7 and 24) as follows:

The detected IS element copies using Southern hybridizations were estimated with the formula:

$$C = \frac{A[ng]*6{,}022*10^{23}[\frac{number}{mol}]}{L[bp]*1*10^{9}\left[\frac{ng}{g}\right]*650[\frac{g}{mol}]}$$

The detected number of *A. asiaticus* cells was calculated with the formula:

$$N = \frac{C}{I}$$

The corresponding detected subpopulation of amoeba hosts was calculated as follows:

$$DS = \frac{N}{5a2}$$

Abbreviations: A (amount of DNA), C (copy number of detected IS elements), DS (detected infected amoeba subpopulation), I (copy number of IS elements in the genome of *A. asiaticus*), L (length of IS element), N (number of detected *A. asiaticus* cells), 5a2 (*A. asiaticus* cells in an infected *Acanthamoeba*). Assumed parameters: average weight of one bp DNA: 650 Da; Avogadro constant: 6.022×10^{23} molecules/mol; sensitivity of Southern blot: 0.1 (detection limit 1 (DL1)) - 0.03 (detection limit 2 (DL2)) pg DNA (according to the manufacturer: Roche); average number of *A. asiaticus* in an infected *Acanthamoeba*: 1000; assumed average length of IS elements: 1000 bp.

Supplementary Table S3: Properties of genes downstream of IS elements

Locus_tag	Best blast hit (BlastP nr) (amino acid identity to best blast hit, GenBank accession no. of best hit)	Putative function (InterPro domain)	Length (aa)	Distance to IS element (bp)	IS element upstream (locus_tag)
Aasi_0071	Putative thioredoxin peroxidase *Algoriphagus* sp. PR1 (67%, ZP_01719713)	Thioredoxin peroxidase (IPR000866)	213	161	ISCaa3 (Aasi_1470/1471)
Aasi_0380	Ferritin like protein 1 *Algoriphagus* sp. PR1 (61%, ZP_01717679)	Ferritin (IPR008331)	175	280	ISCaa8 (Aasi_1564/1565)
Aasi_0449	Sensor histidine kinase/response regulator/transporter hybrid protein *Orientia tsutsugamushi* str. Ikeda (24%, YP_001938332)	Putative Na^{+}/solute symporter (IPR001734, IPR006674)	1126	136	ISCaa6 (Aasi_0448)
Aasi_0800	Sel1 domain protein repeat-containing protein *Haemophilus somnus* strain 2336 (29%, ACA31627)	Tetratricopeptide-like helical (IPR011990)	268	29	ISCaa8 (Aasi_1702/1703)
Aasi_1745*	Hypothetical protein *Gloeobacter violaceus* PCC 7421 (44%, BAC91739)	PilT protein, N-terminal (IPR002716)	132	71	ISCaa16 (Aasi_0897)
Aasi_0969	Viral A-type inclusion protein, putative *Trichomonas vaginalis* G3 (23%, EAX94478)	Hypothetical protein	519	255	ISCaa11 (Aasi_1768)
Aasi_1085	Ankyrin repeat-containing protein, putative *Penicillium marneffei* ATCC 18224 (47%, EEA23321)	Ankyrin repeat (IPR002110)	47	91	ISCaa13 (Aasi_1807/1808)
Aasi_1118	Sel1 domain-containing protein *Magnetococcus* sp. MC-1 (51%, ABK44504)	SEL1 repeat (IPR006597)	329	31	ISCaa6 (Aasi_1117)
Aasi_1135	Protein-L-isoaspartate(D-aspartate) O-methyltransferase bacterium S5 (50%, EFC64543)	Protein-L-isoaspartate(D-aspartate) O-methyltransferase EC:2.1.1.77 (IPR000682)	204	319	ISCaa11 (Aasi_1829)
Aasi_1844	Hypothetical protein ALPR1_08298 *Algoriphagus* sp. PR1 (45%, ZP_01720881)	Conserved transmembrane protein *Bacteriodetes/Chlorobi*	1005	3	ISCaa15 (Aasi_1172)

* We could not show contiguous transcription between Aasi_0897 and Aasi_1745
